# Epidemiological Dynamics of a Visually Apparent Disease: Camera Trapping and Machine‐Learning Applied to Rumpwear in the Common Brushtail Possum

**DOI:** 10.1111/1749-4877.12995

**Published:** 2025-05-28

**Authors:** Elise M. Ringwaldt, Jessie C. Buettel, Scott Carver, Barry W. Brook

**Affiliations:** ^1^ School of Natural Sciences, Biological Sciences University of Tasmania Hobart Tasmania Australia; ^2^ Odum School of Ecology University of Georgia Athens Georgia USA

**Keywords:** brushtail possum, camera trapping, deep learning, disease ecology, rumpwear

## Abstract

Visually apparent diseases are valuable for investigating and monitoring the occurrence and prevalence of pathogens in wildlife populations through passive monitoring methods like camera trapping. Rumpwear, characterized by visible clinical signs of hair breakage and damage on the lumbosacral region, affects common brushtail possums (*Trichosurus vulpecula*) across Australia. However, the etiology of rumpwear remains unclear, and the spatiotemporal factors are understudied. This study investigated the epidemiology of rumpwear in common brushtail possums at Adamsfield, Tasmania (Australia), and predicted rumpwear distribution across the Tasmanian landscape. We visually classified images of rumpwear clinical signs in 6908 individual possums collected from a 3‐year camera trapping network. Our results revealed that: (1) adults were twice as likely to show signs of rumpwear compared to young possums; (2) rumpwear occurrence increased with the relative activity of possums at a site; and (3) prevalence of rumpwear was seasonal, being lowest in May (3.2%—late autumn) and highest in December (27.1%—early summer). Collectively, these findings suggest that the occurrence of rumpwear may be density dependent, the putative etiological agent seems to be influenced by seasonal factors or site use. Additionally, a convolution neural network (CNN) was trained to identify rumpwear automatically based on the manually (human‐expert) classified camera trap images. Applying the trained classifier to 38,589 brushtail possum images from across Tasmania, the CNN predicted that rumpwear is widespread, with an overall prevalence of 18.6%. This study provides new insights into rumpwear epidemiology and identified factors for further investigating within this host–pathogen system.

## Graphical abstract

Visually apparent diseases are valuable for investigating and monitoring the occurrence and prevalence of pathogens in wildlife populations through passive monitoring methods like camera trapping. Rumpwear is characterized by visible signs of hair breakage and damage on the lumbosacral region of common brushtail possums.

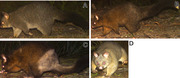

## 1 Introduction

Landscape epidemiology remains understudied in wildlife, partially owing to the logistics of landscape‐scale pathogen surveys, such as live capture and sampling of hosts for pathogens (Morner et al. 2002; Schilling et al. 2022; Stallknecht 2007). However, visually apparent diseases in wildlife offer a valuable opportunity to explore the dynamics of disease‐host systems at both fine and landscape spatial scales. When combined with non‐invasive methods, such as combining visual clinical signs of disease with remote‐sensing techniques like camera trapping, landscape epidemiology research in wildlife can be done more efficiently in terms of resources, compared to methods necessitating capture (Murray et al. 2021; Ryser‐Degiorgis 2013; Schilling et al. 2022). Moreover, landscape epidemiological investigations of visually apparent diseases can also be valuable for understanding dynamics even when the etiology of the visual signs of a disease are unknown, providing a basis for further investigations into causal processes (e.g., Muneza et al. 2019). Examples of such diseases include treponeme‐associated hoof disease in elk (*Cervus elaphus*) (Han et al. 2019; Wild et al. 2022), rumpwear in brushtail possums (*Trichosurus* spp.) (Hufschmid et al. 2010) and ringtail possums (*Pseudocheirus peregrinus*) (Ringwaldt et al. 2022), and skin disease in giraffe (*Giraffa* spp.) (Kalema 1996; Muneza et al. 2016; Whittier et al. 2020).

Rumpwear, also known as “rumpy possum” or “rumpiness” is a visually apparent condition affecting brushtail possums (*Trichosurus* spp.) across Australia (Hufschmid et al. 2010; Munday 1966), and recently identified in the common ringtail possum (*P. peregrinus*) in Tasmania, Australia (Ringwaldt et al. 2022). Mechanisms behind the potential etiology of the visual clinical signs are not well understood but are hypothesized to be caused by hypersensitivity to an irritant, such as an ectoparasite (Munday 1966; Presidente et al. 1982), with associated hair breakage thought to be from mechanical damage, such as overgrooming (Hufschmid et al. 2010). Rumpwear manifests externally as bilateral hair damage to complete hair loss (in rare occurrences) on the lumbosacral region of the possum (Hufschmid et al. 2010; Munday 1966). Ectoparasites such as fleas, *Trichosurolaelaps* spp. of mites, or fur‐clasping mites *Atellana papilio*, have been hypothesized as causal, but evidence is not definitive (Clark 1993; Hufschmid et al. 2010; Munday 1966; Presidente 1978), and more research is needed.

The distinct characteristics of rumpwear have led to investigations into the cause of the condition (e.g., Clark 1993). However, few studies have investigated or identified the environmental or host factors that exacerbate disease occurrence or prevalence over time. Hufschmid et al. (2010) was the first to investigate factors that might influence rumpwear in two brushtail possum species (*Trichosurus vulpecula* and *Trichosurus cunninghami*), finding that rumpwear was common in populations and that demographic factors may influence disease status of individual possums (Hufschmid et al. 2010). Studies have suggested that social stress due to high‐density populations increase prevalence (Reiss et al. 2015; Viggers and Spratt 1995), or severity increases when individuals are immunologically compromised, particularly during the breeding season (April to May) (McKay and Winter 1989). However, studies encompassing variable possum abundance or activity combined with seasonal patterns are yet to be done (Hufschmid et al. 2010).

In this study, we used visual clinical signs combined with longitudinal camera trapping to investigate the epidemiology of rumpwear at different spatial scales in common brushtail possums (*T. vulpecula*) in Tasmania, Australia. Our objectives were to: (1) determine if rumpwear was related to demographic factors, by observing mothers and back‐young with and without rumpwear; (2) determine the relationship between time, climate, habitat, and host variables on the occurrence of rumpwear in individual common brushtail possums across sites in the region of Adamsfield, Central Tasmania; and (3) use the expertly labeled camera trapping images classified from the Adamsfield region to train a deep‐learning image‐classification model, and use this to generate a map of predicted rumpwear occurrence across Tasmania. Our study directly addresses temporal and spatial knowledge gaps identified in the epidemiology of this novel condition and uses advances in machine learning to facilitate bulk image processing and assessment of wildlife diseases.

## 2 Materials and Methods

### 2.1 Study Area and Camera Trapping Networks

**FIGURE 1:**
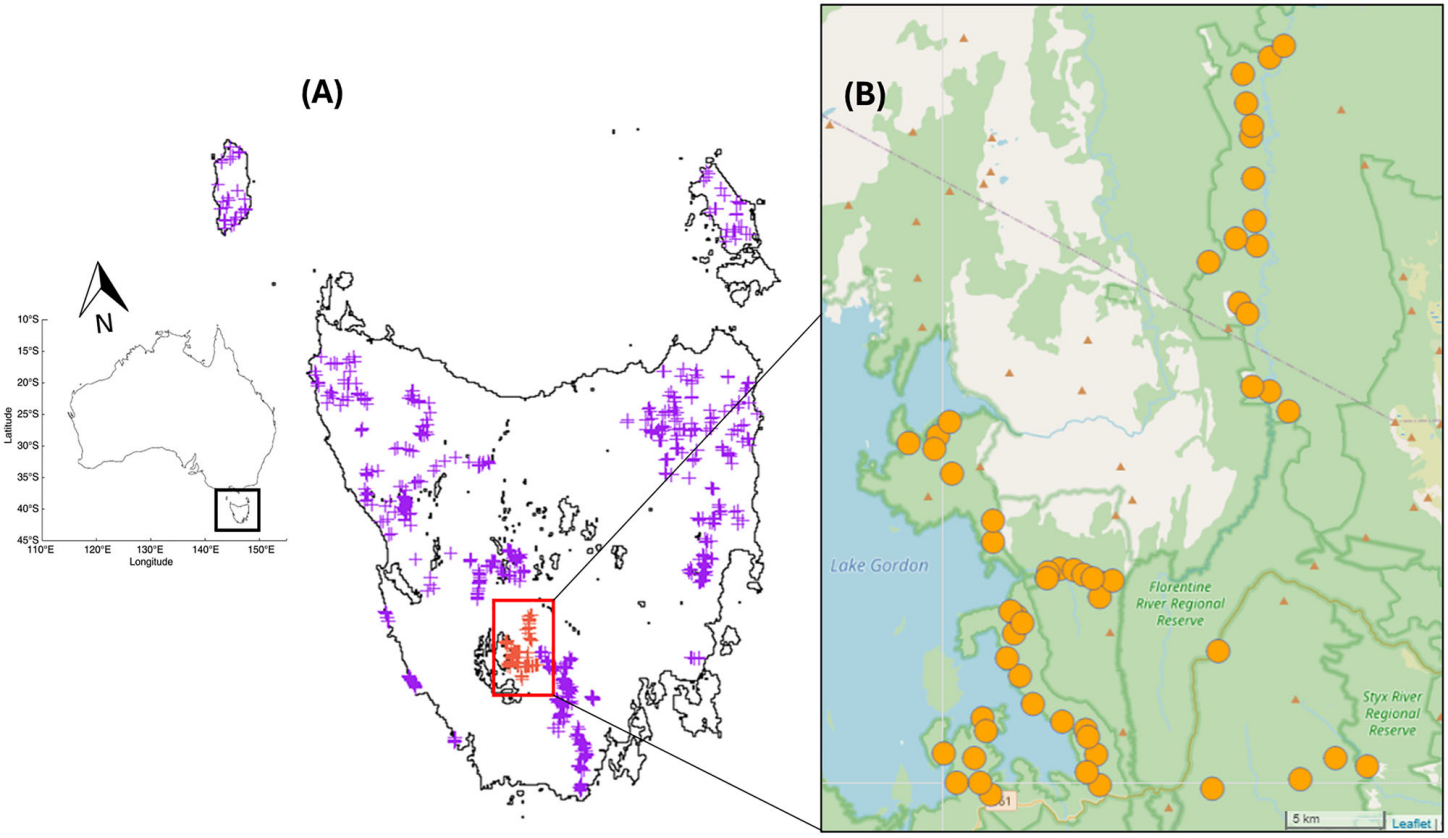
Camera trap locations used to investigate rumpwear in common brushtail possums (*Trichosurus vulpecula*) from Tasmania Australia. (A) Camera trap locations from across Tasmania (landscape scale, 869 camera traps). (B) the Adamsfield region, comprising 57 sites formed by aggregating camera traps to the average home range size of brushtail possums (330 m). Sites in (B) were used for region‐level analyses (AIMS 1 and 2) and the remaining camera trap locations in (A) were used as part of landscape‐level analyses (AIM 3).

We used observational data collected from camera trapping networks placed across Tasmania, Australia (68401 km^2^ island state). Cuddeback X‐change Model 1279 camera traps were placed 100 m or greater apart and were fixed to trees (∼30 cm off the ground) on forestry roads or obvious wildlife tracks. Each camera trap was programed to take one image when triggered by heat and movement with a 30 s wait time between triggers. No baits or lures were used. We used two datasets that represented two contrasting spatial scales: (1) the region of Adamsfield, Tasmania (centroid 42.7333°S, 146.3158°E), for which we had 3 years of common brushtail possum population monitoring from camera traps placed between June 5, 2018 to May 15, 2021; and (2) Tasmanian landscape, with common brushtail possum images collected over a period of November 29, 2016 to March 1, 2022. In total there were 125 camera traps within the Adamsfield region and 744 additional camera sites from across the Tasmanian landscape. Camera traps and associated common brushtail possum images encompassed a state‐wide monitoring network by members of the Dynamics of Eco‐Evolutionary Patterns Group at The University of Tasmania, totaling 869 camera trap locations across the island (Figure 1A, Supporting Information S1).

### 2.2 Adamsfield Study Region and Classification of Images

We aggregated the 125 camera trap locations from the Adamsfield region into “sites” for the purpose of our analyses. Sites were characterized by the aggregation of cameras within 330 m of each other using a hierarchical distance matrix. This aggregation of camera traps resulted in 70 sites which covered a 34‐ha circle. The 330 m distance between sites helped mitigate spatial autocorrelation among observations, as this is generally greater than the nightly movement range of a common brushtail possums (*T. vulpecula*) (Harper 2005); and the squared distance between the center of sites (10.9 ha) corresponds approximately to the average home range (males with 15.5 ha and females with 6.8 ha; Harper 2005; McKay and Winter 1989; Statham and Statham 1997). Of the 70 sites, 13 recorded no possum images and were omitted because they could not contribute to our response variable (presence or absence of rumpwear), leaving 57 sites for the Adamsfield region analyses (AIMS 1 and 2) (Figure 1B). The co‐ordinates (latitude and longitude) of each of these 57 sites were determined by calculating the center point of all camera trap locations within each site.

**FIGURE 2:**
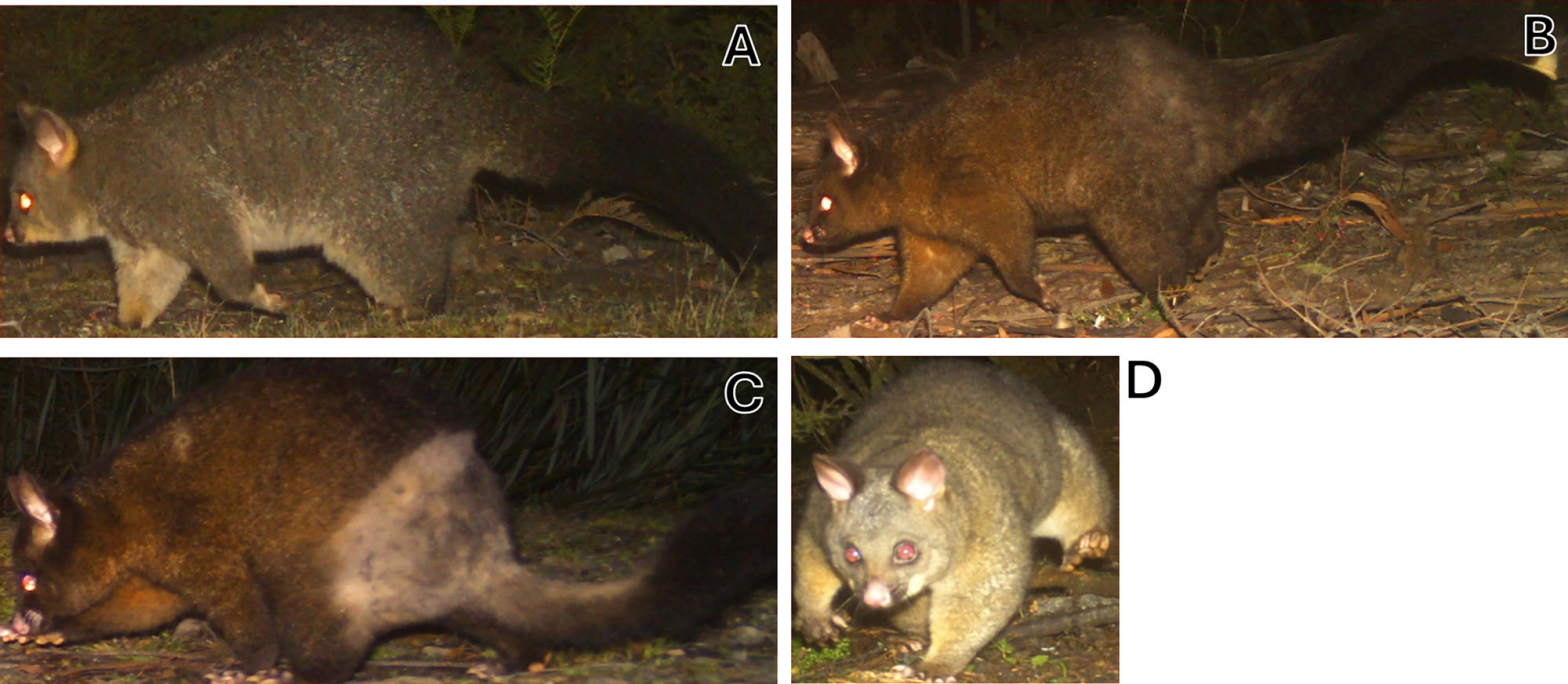
Example images from camera traps showing the stages of rumpwear in brushtail possums (*Trichosurus vulpecula*), manually scored as: (A) Healthy, no rumpwear—class assigned as “0”; (B) Uncertain signs of rumpwear,“1”; (C) rumpwear certainty with clear hair loss and breakage, “2”; and (D) unable to see the rump in the image as it is obscured by vegetation or capture position, “NA.”.

We manually scored every common brushtail possum image for visual clinical signs of rumpwear, using the definition of rumpwear hair loss and breakage described in Hufschmid et al. (2010) and adapted from Lugton (2003). Rumpwear externally presents as bilateral hair damage and breakage affecting the lumbosacral region of the possum and can visually change coat color on the rump to light gray (cream fur) and in some cases complete hair loss can occur (Hufschmid et al. 2010; Munday 1966; Presidente et al. 1982). Rumpwear can also be present with varying degrees of scab formations, and therefore lumbosacral dermatitis has been synonymously used with rumpwear; however, coat damage may be present without dermatitis and therefore ulcerations or dermatitis were not used as an assessment for rumpwear (Hemsley and Canfield 1994; Hufschmid et al. 2010). Rumpwear characteristic scores were assigned on an ordinal scale: 0 = healthy (no signs of rumpwear); 1 = uncertain (ambiguous signs which could be due to fighting or scratching); 2 = rumpwear (early to severe signs of rumpwear: such as missing hair, difference in fur coloration, hair thinning, to complete hair loss in some areas); and, if the lumbosacral region of the possum was not visible, we scored the image as “obscured” and therefore could not be assessed (Figure 2).

Young possums in our study were classed as back‐young visible on mothers, or smaller individuals following an adult (mother) possum. We classed young as dependent, which are different from juveniles (also known as sub‐adults). Juveniles are independent, less than 2 years old and likely to be immature. In contrast, adults are over 2 years and deemed mature (Efford 1998; Webster et al. 2014). In our study we were unable to separate juveniles from adults reliably through camera trap images, and therefore adults in our study likely include both immature and mature possums. All but one young individual was recorded in an image with an adult possum (*n* = 396). From the camera trap images, there were 395 adult and young pairs recorded.

### 2.3 Common Brushtail Possum Relative Activity in the Adamsfield Region

We used the number of trap nights with a possum event across the length of time (unit) as a measure of relative activity (site use). For each of the 125 camera traps, the total number of trap nights over which a camera was operating (effort), and the total number of trap nights recording a possum event (total days present), were summed for each site and month of the year. The proportion of days with a possum event (PDE) was used as a normalized index of “activity” for each month (unit) per site (calculated as: *total days present/effort*). This is a common metric for activity (and site use) used within camera trap studies that corrects for differences in effort (O'Brien 2011; Broadley et al. 2019; Read et al. 2015)

### 2.4 Environmental and Landscape Variables at the Adamsfield Region

We selected general environmental and habitat variables a priori as those “most likely” to influence rumpwear in brushtail possums (Supporting Information S2). We used the Tasmanian Vegetation Monitoring and Mapping Program: TASVEG version 4.0 (TASVEG4 2020) raster at a nominal scale of 1:25,000 (250 m) for data on vegetation type. We re‐classified vegetation type into two categories: “forest” and “non‐forest” (Supporting Information S3). The “forest” category included the vegetation communities of dry and wet eucalypt forest, woodland, and rainforest and related scrub. The “non‐forest” category included heathland moorland, sedgeland, grasslands, and coastal complexes. We used the List Digital Climate Maps of Tasmania produced by the Tasmanian Government for the average annual rainfall (mm), and the mean maximum temperature (°C) for each site (theLIST 2021). Rainfall and temperature data were calculated using a collection of high‐resolution climate grid surfaces, which were averaged to 2018, resulting in spatial resolution of 30 m for temperature and 80 m for rainfall data. We extracted all vegetation and climate data from each layer using the “raster” package's *extract* function in R (Hijmans 2022). Three sites were on the border of waterbodies and therefore the nearest neighbor cell was used to extract all environmental variables.

### 2.5 Statistical Analyses

For spatial autocorrelation analysis and AIM 2, we separated adult possum images into 10‐min intervals for independence of observations of possum and rumpwear “events,” as some individuals occupied multiple 30 s camera triggers; these methods are consistent with standard protocols (e.g., Meek et al. 2014; Thalmann et al. 2014).

Prior to evaluating the relationship of covariates to rumpwear in the Adamsfield region (AIMS 1 and 2), we tested for spatial autocorrelation among sites using Moran's I from the “ape” package (Paradis et al. 2004), and created a distance matrix of each site's latitude and longitude. To meet the assumptions of Moran's I we log‐transformed the number of rumpwear events per site to normalize the data before running analysis. We observed no significant spatial autocorrelation between rumpwear events per site (log) using Moran's I (*p* = 0.202, SD = 0.0384, Supporting Information S4). Thus, we did not consider spatial autocorrelation further in our analyses.

### 2.6 AIM 1: Differences in Rumpwear Between Adults and Young Brushtail Possums

To establish if there was a difference in the occurrence of rumpwear with age (see Section 2.2 for age classification) we used a binomial logistic regression on rumpwear presence (0 or 1, where 1 included both certain and uncertain cases of rumpwear) with age as the predictor variable, no other variables were included. Furthermore, to determine whether young exposed to the rumpwear condition on adults (mothers) were also likely to display clinical signs of rumpwear, a Fisher's exact test was applied. This was used to determine if the rumpwear scores for paired adults and young (*n* = 395) were associated.

We omitted young possums from subsequent Adamsfield region analyses (AIM 2) due to technical non‐independence of data between young and adults—young are either riding on the back of the adult or following closely behind—and because of results found during AIM 1. Outside of young occurrences, possums generally occurred singularly in images, allowing for one rumpwear score per possum image; and the vast majority of possum images were adults (94%, see Section 3). In preliminary analyses we examined if this removal had any impact of AIM 2 findings, showing that there was no effect (Supporting Information S5).

### 2.7 AIM 2: Predictors of Rumpwear in the Adamsfield Region

First, all continuous predictor variables were standardized by centering and dividing them by their respective standard deviations using the R function *scale*. To examine intercorrelation, a Spearman's rank correlation was used, with the finding that all continuous predictor variables were sufficiently independent to be retained (correlation coefficient of <0.7, Supporting Information S6). Next, we explored relationships in our data graphically to determine which, if any, variables had obvious nonlinear relationships with our data, necessitating analytical model consideration, finding this was appropriate for month (see Section 3).

We used an ordinal mixed‐effects generalized additive model (GAM) from the “mgcv” package (Wood 2017, 2021) to accommodate the hierarchical ordering of the rumpwear response variable (i.e., scores of 0, 1, or 2, from each independent possum event) and the linear and nonlinear relationships evident among predictor variables. This modeling approach allowed us to estimate the cumulative probabilities associated with each category while considering their quantitative order. We used the default *theta* = 1, assuming that the relationship between the different categories of rumpwear is the same across the entire range of predictor variables. In our ordinal mixed‐effects GAM, linear predictor variables included average annual rainfall, average maximum temperature, vegetation type, year, and relative activity of possums; a nonlinear predictor variable of month; and site as a random effect. All predictors were continuous variables, except vegetation type and year, which were categorical. All analyses were done in R version 4.1.0 (R Core Team 2021).

### 2.8 AIM 3: Deep‐Learning Classification and Inference of Rumpwear Across Tasmania

To address AIM 3 of our study, we used a convolution neural network (CNN) for automated disease‐state identification from imagery; such an approach has been used recently for human skin diseases (Maduranga and Nandasena 2022). To do this, we leveraged the expert‐tagged possum images from Adamsfield (used in AIMS 1 and 2) and manually classified an additional 2000 images from across the state, to build a rumpwear‐condition classifier for making inferences across the entire Tasmania‐wide camera network.

For each labeled image, the animal was located and isolated using MegaDetector (Beery et al. 2019) and resized to a 384‐pixel square crop. Our final labeled sample consisted of 4022 healthy, 1679 diseased, and 2077 obscured crops. Prior to training, we separated a random 10% of each class for validation and 10% for a test set.

We used the EfficientNet (EN) v2‐M CNN model (Le and Tan 2021), with transfer learning of weights from ImageNet. This was implemented using the Keras TensorFlow API in Python 3.9 (https://keras.io) using focal loss and the Adam optimizer, with image augmentation via the imgaug library (magnitude 22). The model top was replaced with a dropout layer (for regularization, set at 0.2) and two fully connected layers of 512 neurons (compression layer, ReLU activation) and 3 (classification, softmax) neurons. We first trained the top of the model while leaving the other weights frozen. We then made the two uppermost EfficientNet‐v2 blocks trainable and fine‐tuned them with a small learning rate of 1 × 10⁻⁶ (1e − 6), with early stopping assessed against the validation set.

The final model (stopped at 25 epochs) had an overall augmented training loss of 0.0092 and accuracy of 98.5%, and an unaugmented test loss of 0.0178 and accuracy of 98.4%. We used this model to predict the class and disease status of 38,589 other common brushtail possum images from regions across Tasmania, to provide a geographic estimate of rumpwear distribution. For each image the deep‐learning model returns a classification of rumpwear (healthy, rumpwear, and unable to see rump) alongside the probability of that classification. There were 81 cases where two or more common brushtail possums were recorded in one image, however these instances were counted as a single possum observation and the CNN would return the category with the highest probability for that image. The probability reflects the relative likelihood that the image classification belongs in that category, on a scale from 0 to 1.

## 3 Results

### 3.1 AIM 1: Differences in Rumpwear Between Adults and Young Brushtail Possums

In total 6908 common brushtail possums were manually scored for rumpwear from a total of 6478 images, of which 6512 were adults and 396 were young (Table 1). There were 431 images which had multiple animal observations, of these, 467 were adults and 395 were young brushtail possums. Of the possums for which we could see the rump (excluding 506 obscured images), 84% (*n* = 5058) of adults were healthy and 16% (*n* = 948) had rumpwear or uncertain signs. There were 98% (*n* = 312) of young without rumpwear and 2% (*n* = 6) with rumpwear or uncertain signs, with 78 obscured images excluded (Supporting Information S7).

**TABLE 1:** All common brushtail possum adult (mother) with young (dependent) pairs recorded within the same image and the corresponding rumpwear observations.

Pairs (young) (adult)	Healthy	Uncertain	Rumpwear	Obscured	Total
**Healthy**	226	2	0	52	280
**Uncertain**	32	1	0	7	40
**Rumpwear**	20	0	2	2	24
**Obscured**	33	1	0	17	51
Total	311	4	2	78	395

We found statistical evidence that rumpwear (both certain and ambiguous signs) was associated with life stage (LRT, G1 = 67.47, *p* < 0.001), where young brushtail possums were less likely to have rumpwear than adults (−2.28, 95% CI [−3.208, −1.557]).

We performed a Fisher's exact test on adult and juvenile pairings and found a significant association between the rumpwear score category of adults and juveniles (*p* = 0.005). Of the six young with rumpwear or ambiguous signs, only three adult pairings had the same rumpwear score (Table 1).

### 3.2 AIM 2: Predictors of Rumpwear in the Adamsfield Region

**FIGURE 3:**
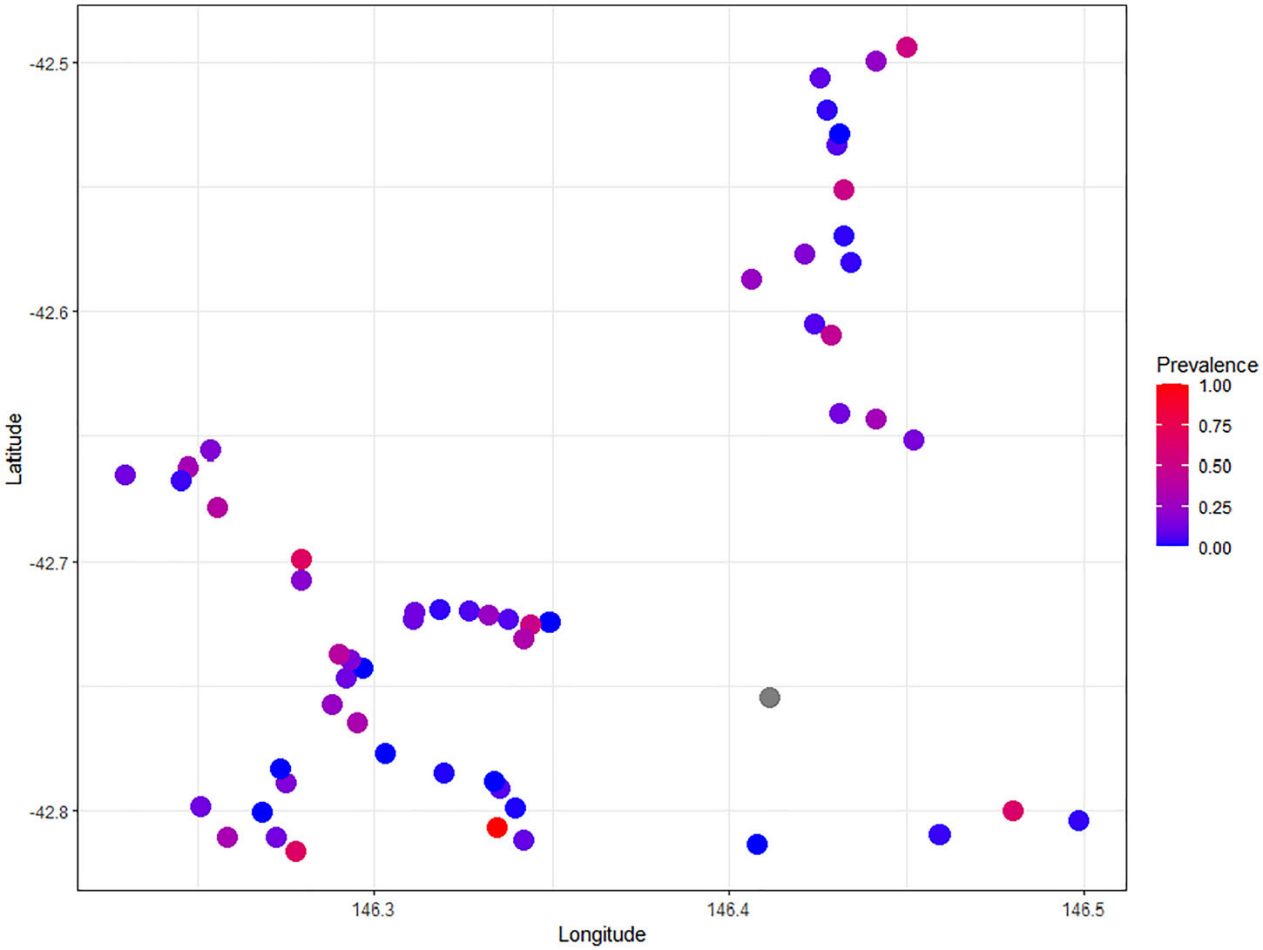
The proportion of rumpwear at each site in the Adamsfield region (scale, from blue [0.0] to red [1.0]). The proportion of common brushtail possums with rumpwear included both rumpwear certain and uncertain categories (calculated: possums with rumpwear/total possums able to be assessed). It shows there is variation in the proportion of possums with rumpwear across sites. One site (gray) only had one possum present and its rump was obscured, so no assessment could be made.

**FIGURE 4:**
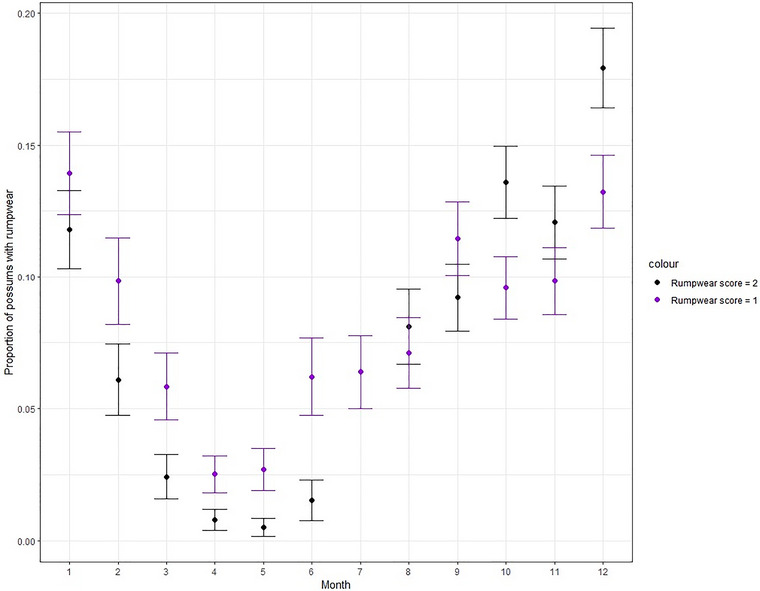
The proportion of rumpwear at Adamsfield for each month over all years (standard error bars, covering the period 2018–2021). The proportion of common brushtail possums with rumpwear for both categories: rumpwear certain (rumpwear score = 2) and uncertain (rumpwear score = 1) (calculated: *possums with rumpwear (1 or 2)/(total possums assessed—possums scored in the other rumpwear category*)). This figure shows both rumpwear categories follow similar trends over the year. July (month 7) has equal proportion for both rumpwear scores. The temporal trend (including both certain and uncertain) shows the Autumn months (months 3–5) having lower prevalence of rumpwear (4.5%) in brushtail possums than other seasons (spring [19.9%], summer [23.0%], and winter [(11.6%]).

We retained 6267 individual adult possum events after excluding all non‐independent observations. Of the 5792 adult possum events from which we could see the rump (475 were obscured and excluded from the analyses, from the original 6267 events), 84% (*n* = 4870) were healthy, 8% (*n* = 461) were uncertain, and 8% (*n* = 461) exhibited rumpwear. Using an ordinal mixed‐effects GAM we found that rumpwear frequency varied among sites (Table 2, Figure 3, Supporting Inforamtion S8). Study year and the relative activity of brushtail possums were significant linear predictors of whether an individual possum had rumpwear (Table 2, Supporting Information S8). Rumpwear occurrence in brushtail possums was similar from 2018 to 2020 but greater in 2021 compared to 2018 (0.44, 95% CI [0.012, 0.860]; *p* = 0.044), and higher activity of possums was positively associated with rumpwear (0.137, 95% CI [0.037, 0.238]; *p* = 0.007). Month was a significant nonlinear predictor of rumpwear in possums (Table 2, Figure 4). April (3.3%) and May (3.2%) had a lower prevalence compared to other months, particularly December (27.1%) and January (22.8%). These monthly trends were consistent over the 3‐year study period (see Supporting Information S9). Aggregating rumpwear prevalence into seasons, we observed that autumn (March to May, 4.5%) had a lower prevalence of rumpwear compared to summer (December to February, 23.0%) (Supporting Information S9). There was no relationship of rumpwear to rainfall, temperature and vegetation type (Table 2).

**TABLE 2:** The ordinal mixed‐effects GAM test statistics and *p*‐values for the predictor variables on the occurrence of rumpwear in adult common brushtail possums. Showing the degrees of freedom (df), Chi square value (χ²), and *p*‐value for the linear predictors; and the effective degrees of freedom (Edf), reference degrees of freedom (Ref.df), χ², and *p*‐value for random and spline effect variables. See Supporting Information S8 for each individual year coefficient estimates, confidence intervals, and *p*‐values.

Predictor variables		df	χ²	*p*
Rainfall Temperature Vegetation Year Relative activity		1	0.044	0.834
		1	0.704	0.401
		1	0.074	0.786
		3	5.801	0.122
		1	7.228	**0.007**
	**Edf**	**Ref.df**	**χ²**	*p*
Month (spline)	6.865	7.923	228.2	**<0.001**
Site (random effect)	42.78	52.00	815.7	**<0.001**

### 3.3 AIM 3: Deep‐Learning Classification and Inference of Rumpwear Across Tasmania

CNNs trained for rumpwear in common brushtail possums classified 38 589 images, totaling 46 367 possum images scored for rumpwear across the 869 Tasmanian camera traps (Table 3). Among these observations, 28 721 were classified as healthy, 8513 as having rumpwear, and 9133 as obscured (Table 3).

**TABLE 3:** The total number of common brushtail possum images classified by convolution neural networks for each of the three classes (healthy, rumpwear, and obscured) and the average probability score (between 0 and 1) and standard error for each.

	Healthy	Mean probability (±SE)	Rumpwear	Mean probability (±SE)	Obscured	Mean probability (±SE)	Total
Adults	27 331 (61.7%)	0.815 (±0.002)	8226 (18.6%)	0.796 (±0.004)	8770 (19.8%)	0.813 (±0.004)	44 327
Juveniles	1390 (68.1%)	0.768 (±0.011)	287 (14.1%)	0.741 (±0.026)	363 (17.8%)	0.830 (±0.020)	2040

**FIGURE 5:**
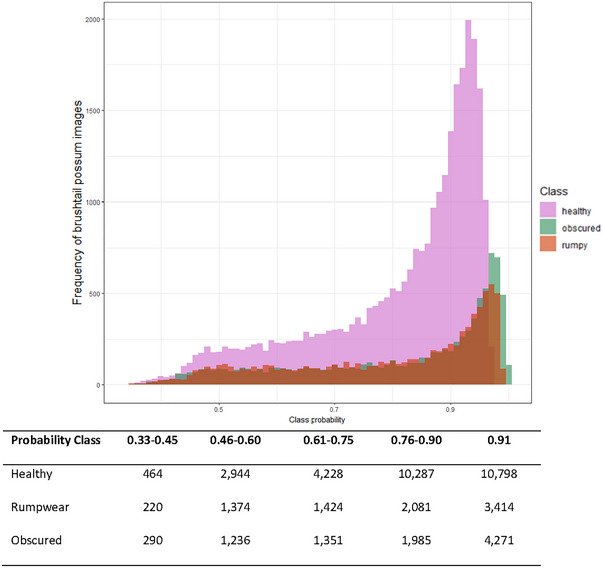
Frequency distribution of CNN‐predicted probability for each class (healthy, rumpwear, and obscured). The lowest probability assessment given to any image in a class was 0.33 (33%). The dataset was split into the probability classes of low (0.33–0.45), low–medium (0.46–0.60), high–medium (0.61–0.75), high (0.76–0.9), and very high (0.91) confidence.

**FIGURE 6:**
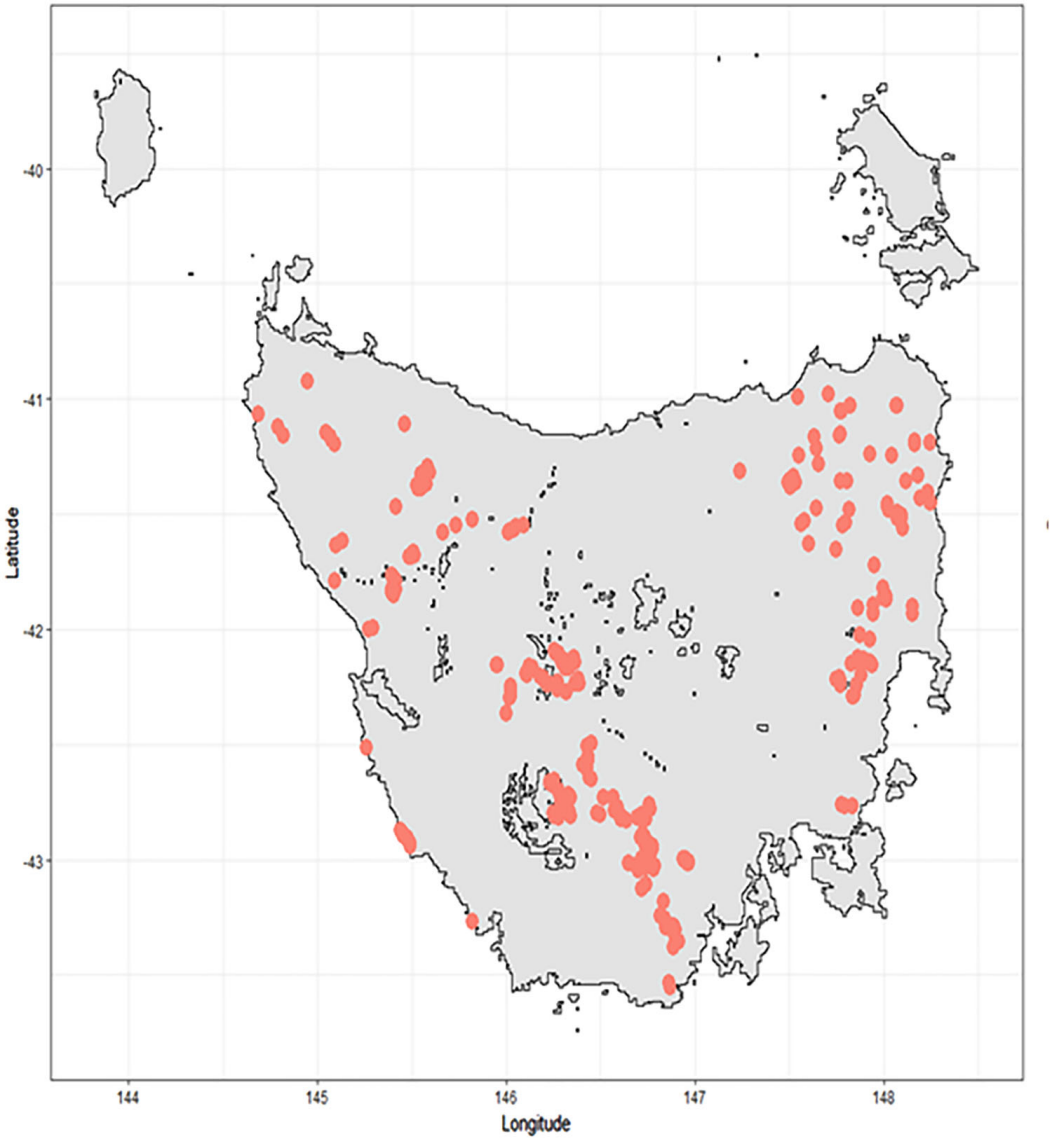
Distribution of common brushtail possums predicted to have rumpwear with very high confidence. Locations of the 3414 images classified above 0.91 probability of rumpwear by the convolution neural network (CNN) are shown as orange circles across Tasmania, Australia. The spatial distirbution indicates that rumpwear is predicted to occur throughout the  Tasmanian mainland distribution of brushtail possums.

We assessed the CNN probability output for each class (healthy, rumpwear, and obscured). The lowest probability assessment possible for any image in a class was 0.33 (33%, where all classes are equally likely). We split the probability assessments into “confidence” categories of low (0.33–0.45), low–medium (0.46–0.60), high–medium (0.61–0.75), high (0.76–0.9), and very high (0.91) (Figure 5). We found the total number of images within a probability class increased as the confidence increased (Figure 5). Furthermore, if the probability cut of “very high” was used as the rumpwear presence only, we can be confident with using this output of the rumpwear distribution across the Tasmanian landscape for strong inference on its geographical distribution. Using the very high probability category, it was evident that rumpwear was found across the brushtail possum distribution in Tasmania (Figure 6).

## 4 Discussion

Using a visually apparent wildlife disease, our study has been able to demonstrate variable, environmentally mediated epidemiology, enhancing insights into patterns of rumpwear in brushtail possums. Our findings suggest that rumpwear occurs across the distribution of common brushtail possums in Tasmania (Southern Australia), with site, time, and host factors driving the prevalence of this condition at regional levels. In particular, rumpwear was more prevalent among adults than young, increased with the relative activity of possums and exhibited seasonal and interannual variation. Collectively, the analysis shows that the occurrence of rumpwear may be density dependent (as indicated by camera activity levels) and the putative etiological agent might have a cyclic temporal pattern, or seasonal activities of possums at a site might also influence the occurrence of disease.

Previous research on rumpwear in possum populations has been limited to infer temporal patterns at regional scales, by focusing on individual sites with smaller sample sizes (Hufschmid et al. 2010). Furthermore, it is possible that seasonal patterns in rumpwear might be harder to detect at individual sites, as in Hufschmid et al. (2010). However, when analyzed across a broader spatial and temporal scale, as in our study, these patterns become more evident. In our 3‐year study, we found that rumpwear has an annual cycle with a lower prevalence during autumn months. Such findings might suggest monthly changes in rumpwear attributed to the life cycle of the putative parasite, the seasonal rhythms of possum life history and habitat quality, or a combination of all (Altizer et al. 2003; Hawley and Altizer 2011; Marquès Gomila et al. 2023). It is well known that parasites are influenced by seasonality and can show yearly trends in disease prevalence and infection rates within their hosts (Altizer et al. 2006; Bethge et al. 2023; Turner and Getz 2010). Changes in climatic and environmental conditions throughout seasons can influence the life cycle of parasites, such as free‐living nematodes (Turner and Getz 2010), mycoplasmal bacteria (Hosseini et al. 2004), tapeworms (Bryan et al. 2012; Miterpáková et al. 2006), and ectoparasites, such as mites (Godfrey et al. 2008; Viljoen et al. 2011). However, in contrast to many other host–pathogen systems (Altizer et al. 2006; Hawley and Altizer 2011; Turner and Getz 2010), we did not find an association between rumpwear occurrence and temperature or rainfall, suggesting climatic variables per se are not as important as other predictive variables within the regional level analysis we undertook for this host–pathogen system.

Monthly changes of rumpwear prevalence in brushtail possums might be attributed to hormonal factors such as stress, or fluctuations in life‐history activity patterns throughout the year (Efford 1998; Hufschmid et al. 2010, 2014). In fact, disease severity can also be exacerbated by a weakened immune system or hormonal stressors (Altizer et al. 2006, 2003; Hawley and Altizer 2011). This phenomenon has been observed in other species such as fossa (*Cryptoprocta ferox*), where alopecia occurs during the breeding season (March to May) (Ratliff and Sutherland‐Smith 2017), and in domestic ferrets, where “estrogen‐induced alopecia” is mainly associated with females and reproductive activity (Bakthavatchalu et al. 2016). Seasonal hair loss has also been reported in, mainly female, Andean spectacled bears (*Tremarctos ornatus*) (Drake et al. 2017; Van Horn et al. 2019). However, in these cases, seasonal hormonal changes and associated hair loss typically occur in females during the breeding season. In our system, common brushtail possums typically breed during the autumn months when rumpwear was significantly lower, although they can breed year round (Efford 1998; Statham and Statham 1997). Moreover, both sexes are known to exhibit rumpwear in brushtail possums, with some studies reporting higher prevalence in females (Lugton 2003), while others have found no difference (Hufschmid et al. 2010; Presidente et al. 1982). Nevertheless, Hufschmid et al. (2014) found seasonal variations in serum cortisol levels, as an indicator of stress, among *T. cunninghami*. They noted that the highest cortisol levels were observed during winter and spring, with females generally exhibiting higher levels. This coincides with the timing of the initial increase in rumpwear prevalence observed in our study, suggesting a delay between the elevation of stress hormones and the development of visual clinical signs of rumpwear, which was most prevalent in summer (December).

We observed differences in rumpwear between adult and young possums, with young less likely to have rumpwear. These findings are consistent with previous studies which have found higher rumpwear or ectoparasites with the increasing age of the possum (Hufschmid et al. 2010; Stankiewicz et al. 1996; Webster et al. 2014), and lower cases of *Mycobacterium bovis* (bovine tuberculosis) infection in juvenile possums (Tobajas et al. 2024). Since the putative agent of rumpwear is believed to be an ectoparasite, the mechanism behind the observed increase could be due to the exposure to parasites with increasing possum age (e.g., Cross et al. 2009; Samuel and Trainer 1972; Slowinski et al. 2022). However, it is also possible that age‐related rumpwear might be socially stress‐induced as possums transition from young to sub‐adult (Hufschmid et al. 2010; Presidente et al. 1982). Social‐stress‐induced alopecia has been observed in other wildlife, such as free‐ranging Formosan macaques (*Macaca cyclopis*) which potentially compounded with immunosuppression (Chen et al. 2021). The maturation of hosts (from young to adults) can also increase the pool of susceptible naïve hosts at certain times of the year, increasing disease prevalence (Altizer et al. 2006; Turner and Getz 2010). This may explain why rumpwear increased during spring and summer, coinciding with young possums maturing into sub‐adults. We also found that the few back‐young possums which had rumpwear signs also had mothers with rumpwear signs, however this was only a small sample size and needs further investigation.

Our study revealed that site‐specific factors play a crucial role in determining the prevalence of rumpwear among brushtail possums at a regional level. Site level complexities have also been documented in other visually apparent diseases (e.g., Caldwell et al. 2016; Chen et al. 2021; Murray et al. 2021), and site‐specific habitat characteristics have strongly influenced other pathogens in brushtail possums, such as bovine tuberculosis (Caley et al. 2001). Although vegetation type (whether the site was forested or not), was not found to be an important influence on rumpwear in our study, the fact that there was distinct variation across sites in rumpwear prevalence suggests that other fine‐scale site‐related differences (that we did not measure) could be important, such as vegetation quality or structure of the habitat at each site (Bethge et al. 2023; Hufschmid et al. 2010; Sousa and Grosholz 1991). For brushtail possums, habitat quality might influence the health of individuals, resources for female to breed, or the density of possums due to nutrient availability (Barnett et al. 1979; Martin and Martin 2007; Presidente et al. 1982). Therefore, to gain a deeper understanding of the site‐specific factors that contribute to rumpwear, detailed characteristics of vegetation type and structure should be considered in future studies. Measuring these local details could provide valuable insights into the site‐specific factors that contribute to rumpwear in brushtail possum populations.

We found that a greater possum activity at a site was a significant contributor to its brushtail possums having higher rumpwear prevalence, supporting observations from previous studies (see Hufschmid et al. 2010; Munday 1978; Reiss et al. 2015). Many pathogens are influenced by host density and abundance, with the pattern being that as transmission rates increase between individuals so can disease prevalence (e.g., Altizer et al. 2003; Arneberg et al. 1998). Comparably, increased movement of hosts, higher host density, and competition for resources can increase stressful environments and indirectly influence the transmission rates of pathogens in wildlife populations (Altizer et al. 2003; Cross et al. 2009). More specifically, greater activity of possums could increase rumpwear occurrence through transmission of the etiological agent through higher contact rates among individuals; increased stress and diminished immunity due to high density of individuals; or a synergy of all, including habitat quality (Altizer et al. 2003; Bethge et al. 2022). Note that rumpwear in our study corresponds to the independence of young to juveniles (around late‐spring to mid‐summer) and time of dispersal of sub‐adults maturing in a population (Hufschmid et al. 2010; Presidente et al. 1982); this could in turn increase possum activity or site densities, increasing social stresses (Reiss et al. 2015; Viggers and Spratt 1995). To gain better insight into the underlying transmission dynamics of the disease, future studies could concentrate on estimating possum density or abundance at sites and measure behavior changes such as variations in contact rates among individuals over time in relation to rumpwear development in individuals.

Using a CNN computer‐vision approach to classify rumpwear, we revealed that rumpwear was a common condition in adult common brushtail possums at both a regional and landscape scale (8%–16% and 19%, respectively). Interestingly, rumpwear prevalence in common brushtail possums was comparable to that observed in the common ringtail possum (*P. peregrinus*), which was recorded at a prevalence of 7% –14% across Tasmania (Ringwaldt et al. 2022). To have confidence in the rumpwear distribution predicted by the CNN, we implemented a cut‐off probability estimate. This approach was effective in our rumpwear system because of the combination of the large number of possum images and a good sample size of expertly labeled data to train the model. Other disease systems which have used CNN or deep learning include agricultural landscapes (e.g., coffee leaf disease; Esgario et al. 2022; Novtahaning et al. 2022) and healthcare (e.g., skin disorders in humans; Kalaivani and Karpagavalli 2022; Maduranga and Nandasena 2022), however using CNN for classification of images in wildlife disease is a new application. To gain a more comprehensive understanding of disease‐dynamics operating at varying temporal and spatial scales, it is crucial to incorporate novel methods for evaluating disease prevalence and distribution in wildlife populations, particularly when dealing with high‐volume data and visual clinical signs of disease.

By combining camera trapping and convolutional neural network approaches, we were able to gain a deeper understanding of both the temporal dynamics and spatial distribution of this disease system. While we refer to rumpwear as a disease in this manuscript, which implies a common etiological agent, it is unknown if more than one etiology causes similar visual clinical signs in possums and therefore, rumpwear should be considered as a syndrome. Additionally, we acknowledge that the inclusion of uncertainty within the analyses, especially when comparing young and adults, risks overestimating the occurrence of the syndrome in possums. Although a conclusive cause of rumpwear cannot be determined from image data, our findings provided important lines of evidence that suggest potential mechanisms and avenues for future research. Since our study was limited to a temperate climate system, further exploration of these mechanisms across the wider continental distributional range of common brushtail possums is necessary, accompanied by fine‐scale studies at site levels. Additionally, the CNN results can be used to assess the landscape‐scale distribution and drivers of rumpwear in possums, and the CNN methods can be applied to other disease‐host systems with labeled images of visually apparent diseases. Our study emphasizes the effectiveness of using visual clinical signs of disease to evaluate prevalence and distribution in wildlife populations, overcoming limitations in sampling pathogens at different spatial scales. Furthermore, it demonstrates that deep‐learning techniques have the advantage of detecting and classifying visually apparent diseases from large datasets, providing a promising tool for future investigations of host–disease relationships.

## Conflicts of Interest

The authors declare no conflicts of interest.

## Supporting information



Supplementary materials

## Data Availability

All data needed to evaluate the conclusions are presented in the manuscript and/or the Supporting Information.
