# Supplementary material for: Epidemiological Dynamics of a Visually Apparent Disease: Camera Trapping and Machine‐Learning Applied to Rumpwear in the Common Brushtail Possum
Source: Integr Zool. 2025 May 28;21(1):116–28. doi: 10.1111/1749-4877.12995 (PMC12794789; doi:10.1111/1749-4877.12995)
Supplement: Supplementary file 1 — Supplementary materials [file INZ2-21-116-s001.docx]

Supplementary materials – Appendix

**1 | Tasmanian wide camera trapping networks.**

In total there were 125 camera-trap locations within the Adamsfield region that were included in the site analyses, no baits or lures were used. For the Tasmania wide analysis, we used brushtail possums images collected from a state-wide monitoring network by members of the Dynamics of Eco-Evolutionary Patterns Group at The University of Tasmania, totalling 869 camera-trap locations. Two networks used baits and lures, those on the Bass Strait Islands and some parts of the Tasmanian south (Figure 1A)).


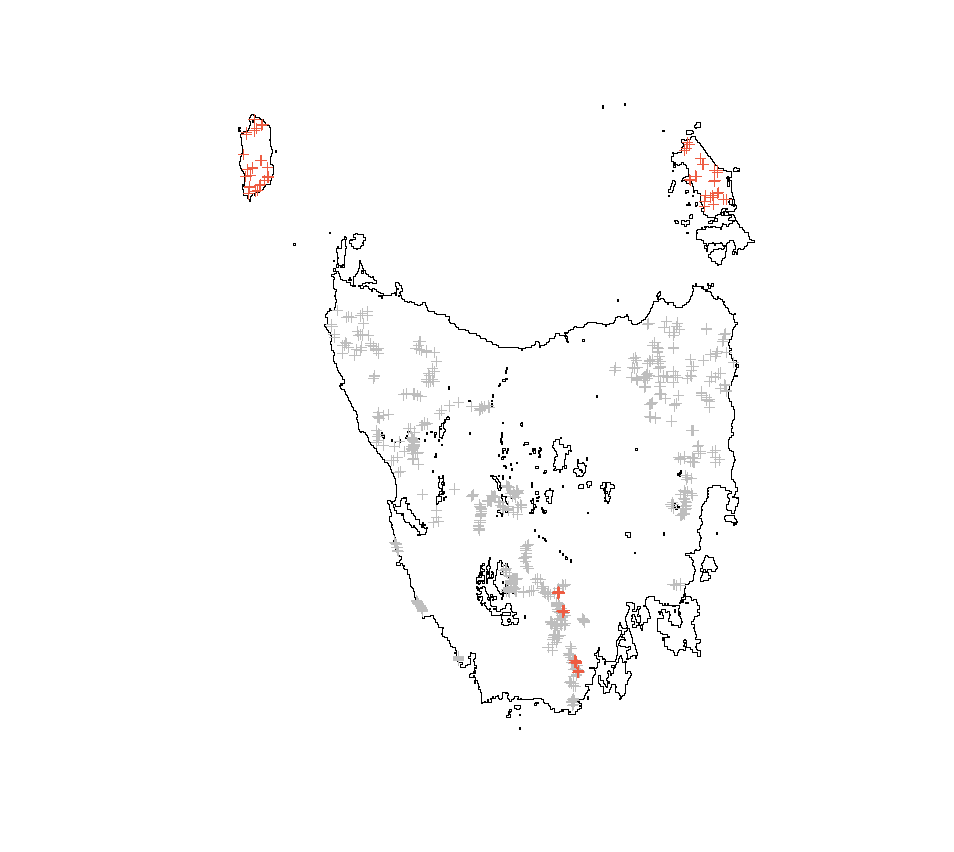

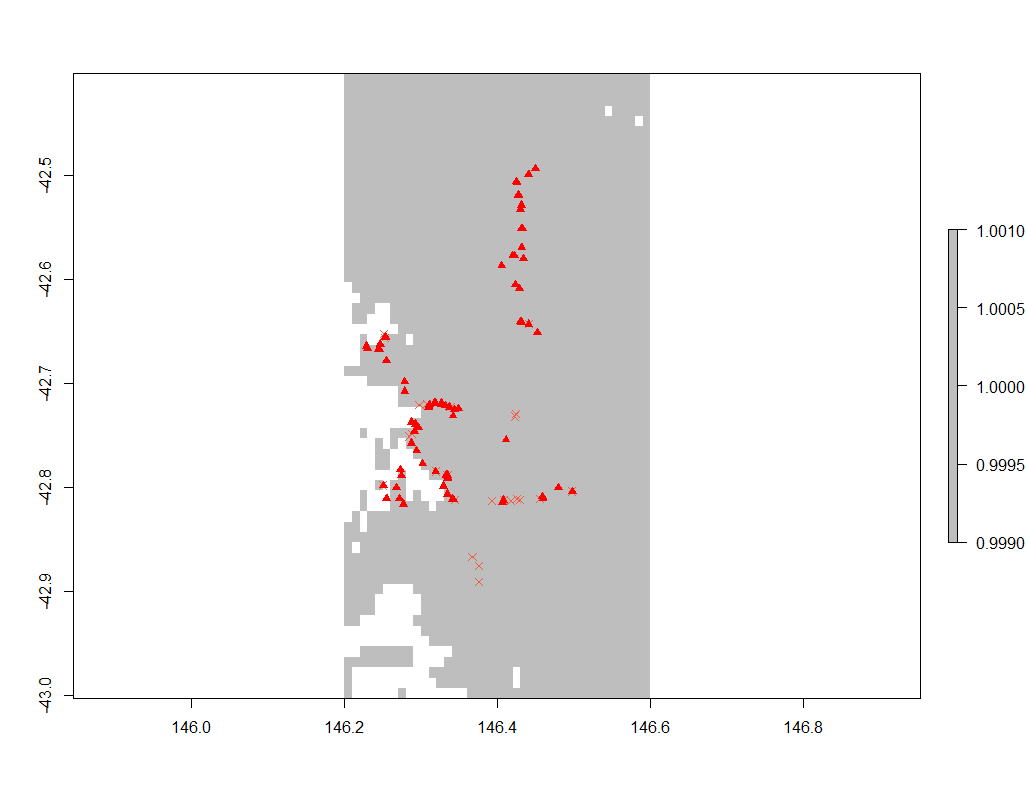

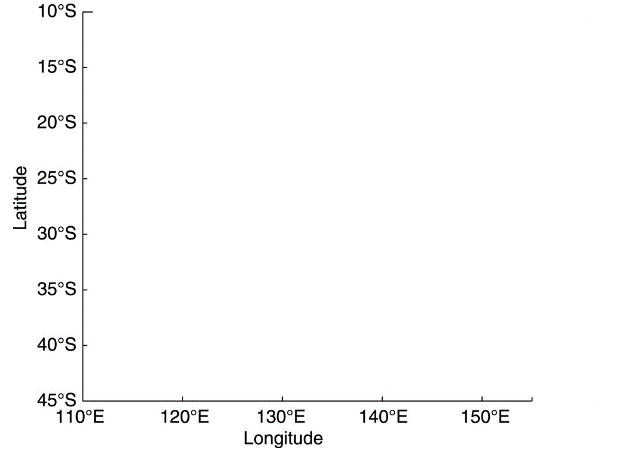

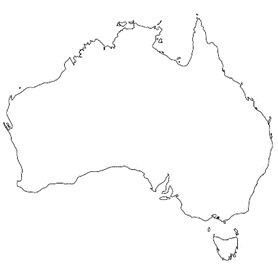

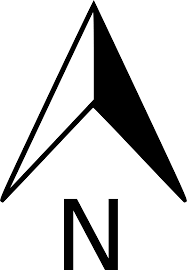


**Figure 1A:** Tasmanian-wide camera-trap sites which included a brushtail possum (*Trichosurus vulpecula*) presence: **(A)** landscape-scale camera-trap sites recording a brushtail possum presence across Tasmania, consisting of 869 camera-trap sites (grey crosses are networks using no baits or lures and red crosses are networks using baits or lures); and **(B)** all camera-trap sites from Adamsfield, containing 101 brushtail possum presence (red triangles) and 24 absence (red crosses) cameras (grey is land and white is fresh water).

**2 | Environmental, climate and host variables likely to influence rumpwear in brushtail possums.**

| **Predictor variables** | **Data source & classification** |
| --- | --- |
| **Rainfall**  Average annual rainfall at the site (averaged at 2018).  Millimetres (mm). | List Digital Climate Maps of Tasmania (raster layers) produced by the Tasmanian Government for the average annual rainfall (mm), (https://www.thelist.tas.gov.au/app/content/data/geo-meta-data-record?detailRecordUID=ba62f124-5906-4471-a01c-9b57b6142055).  The rainfall data was calculated using a collection of high-resolution climate grid surfaces, which were averaged to 2018, resulting in spatial resolution of 80m (numerical) |
| **Temperature**  Average maximum temperature at the site (averaged at 2018).  Degree Celsius (C) | List Digital Climate Maps of Tasmania (raster layers) produced by the Tasmanian Government for the mean maximum temperature (Degree Celsius, C) (<https://www.thelist.tas.gov.au/app/content/data/geo-meta-data-record?detailRecordUID=ba62f124-5906-4471-a01c-9b57b6142055>).  The temperature data were calculated using a collection of high-resolution climate grid surfaces, which were averaged to 2018, resulting in spatial resolution of 30m for temperature (numerical) |
| **Vegetation**  (Reclassified to 2 levels)   1. Forest 2. Non-forest   See below. | Department of Primary Industries, Parks, Water and Environment. TASVEG 4.0, Released July 2020. Tasmanian Vegetation Monitoring and Mapping Program, Natural and Cultural Heritage Division. Downloaded from [www.theLIST.tas.gov.au](http://www.theLIST.tas.gov.au)  (factor) |
| **Relative activity**  The proportion of days with a possum event **(PDE)** for that site per month. | All days recording a possum event from camera traps / total days running for camera traps (numerical) |
| **Site** | All camera traps within 330m of each other (factor) |
| **Year** | Year of the study (factor) |
| **Month** | Month of the study (numerical) |

**3 | Tasmanian Vegetation Monitoring and Mapping Program (TASVEG4) and rasterization for analysis.**

We re-sampled all vegetation communities to the vegetation types of ‘forest’ and ‘non-forest’ categories. The ‘forest’ category included the vegetation communities of dry and wet eucalypt forest, woodland, and rainforest and related scrub. Where-as ‘non-forest’ included heathland moorland, sedgeland, grasslands and coastal complexes. Of the 57 sites, 49 sites were in the forest type, 8 were in the ‘non-forest’ type. Three sites were on the border of waterbodies (group ‘Other (O)’) and therefore the nearest neighbour cell was used to extract all environmental variables.

| **All TASVEG 4.0 Vegetation communities (codes)** | **Major vegetation community** | **Re-sampled to one of two groups (F) Forest or (N) Non-Forest** |
| --- | --- | --- |
| AAP, AHF, AHL, AHS, ARS, ASF, ASP, ASS, AUS, AWU | Saltmarsh and wetland | N |
| DAC, DAD, DAM, DAS, DAZ, DBA, DCO, DCR, DDE, DDP, DGL, DGW, DKW, DMO, DMW, DNF, DNI, DOB, DOV, DOW, DPD, DPE, DPO, DPU, DRI, DRO, DSC, DSG, DSO, DTD, DTG, DTO, DVC, DVF, DVG | Dry eucalypt forest and woodland | F |
| FAC, FAG, FMG, FPE, FPF, FPH, FPS, FPU, FRG, FSM, FUM, FUR, FWU | Modified land | N (FAC, FAG, FMG, FPE, FPF, FRG, FSM, FUM, FUR, FWU)  F (FPU, FPH, FPS) |
| GCL, GHC, GPH, GPL, GRP, GSL, GTL | Native grassland | N |
| HCH, HCM, HHE, HHW, HSE, HSW | Highland treeless vegetation | N |
| MBE, MBP, MBR, MBS, MBU, MBW, MDS, MGH, MRR, MSW | Moorland, sedgeland and rushland | N |
| NAD, NAF, NAL, NAR, NAV, NBA, NBS, NCR, NLA, NLE, NLM, NLN, NME | Native grassland | N |
| OAQ, ORO, OSM | Other natural environments | O (NA) – nearest neighbour |
| RCO, RFE, RFS, RHP, RKF, RKP, RKS, RKX  RML, RMS, RMT, RMU, RPF, RPP, RPW, RSH | Rainforest and related scrub | F |
| SAL, SBM, SBR, SCA, SCH, SCL, SED, SHS, SHW, SKA, SLG, SLL, SLS, SMM, SMP, SMR, SRE, SRF, SRH, SSC, SSK, SSW, SSZ, SWR, SWW | Scrub, heathland and coastal complexes | N |
| WBR, WDA, WDB, WDL, WDR, WDU, WGK, WGL, WNL, WNR, WNU, WOB, WOL, WOR, WOU, WRE, WSU, WVI | Wet eucalypt forest and woodland | F |

**4 | Spatial autocorrelation methods between Adamsfield sites for the predictor variable (rumpwear).**

Of the 57 sites, one site recorded only one possum with ‘obscured’ rumpwear, 48 had rumpwear present at least once over the study period, while 8 never recorded rumpwear. Due to obscured/not able to see rump, we removed all NAs from the dataset to analyse the rumpwear prevalence (creating 56 sites). We used Moran’s I to ensure spatial independence of the total rumpwear per site. To meet the assumptions of Moran’s I we log transformed the number of rumpwear events per site to ensure they were normally distributed before running analysis. Based on the observed versus expected results, we can accept the null hypothesis and confirm there is no spatial autocorrelation between rumpwear counts at each site (p > 0.05).

**Table 4A:** The output of Moran’s I results to ensure spatial independence of rumpwear (log) between sites. Showing the observed and expected, and p value.

| **Morans’ I** | **Rumpwear(log)** |
| --- | --- |
| Observed | 0.03073357 |
| Expected | -0.01818182 |
| SD | 0.03840431 |
| P value | 0.2027714 |


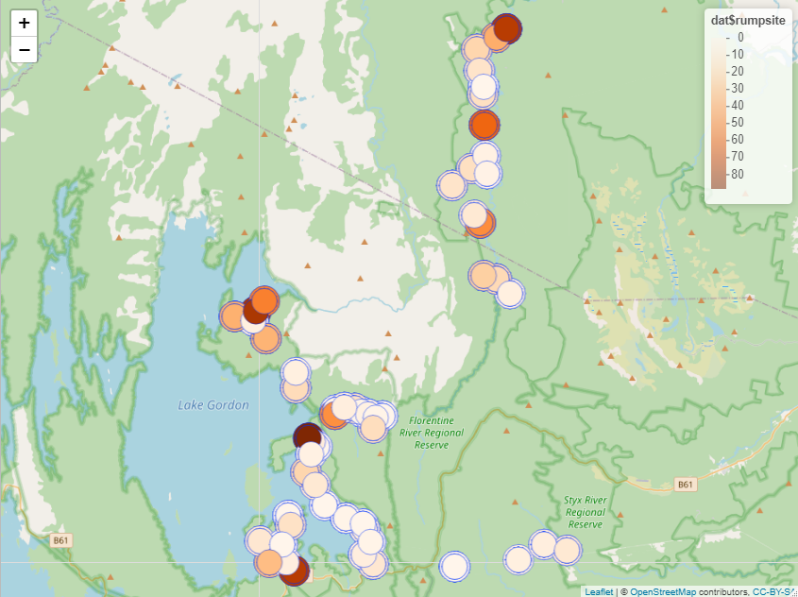


**Figure 4A:** Showing the rumpwear (log transformed) values for each of the 56 sites across Adamsfield region.

**5 | Preliminary analyses of GAM which included young.**

The analyses focused on the non-biased ‘events’ of possums, however, doing so would exclude all young from the analyses. We separated the dataset into adults and young and corrected for events for both datasets. There were 313 young possum events added to the dataset. For the preliminary analyses we combined both adults and young in the ordinal generalised additive models and added life stage as a subsequent predictor.

*GAM: Individual possum event (rumpwear = 0/1/2) ~ average annual rainfall + average maximum temperature + vegetation + year + site activity + life stage + month (numerical spline) + site (random factor)*

We found that by including the young events in the generalised additive models there was no major differences in the outcome and interpretation of the results (compared to using adult possums only). Again, we found that life stage had a significant influence on the occurrence of rumpwear in brushtail possums, with young less likely to have rumpwear than adults (-3.03, 95%CI = -3.862, -2.195; SE = 0.425; P<0.001)

| ***Predictor variables*** | **Estimate** | **Std. Error** | **z value** | **CI, 5** | **CI, 95** | **P** |
| --- | --- | --- | --- | --- | --- | --- |
| Intercept | - 3.06671 | 0.25095 | -12.221 | -3.55855478 | -2.574867465 | < 0.001 |
| Rainfall | 0.05450 | 0.26012 | 0.210 | -0.45531947 | 0.564329145 | 0.83403 |
| Temperature | -0.18981 | 0.22781 | -0.833 | -0.63630711 | 0.256683558 | 0.40473 |
| Vegetation (N) | 0.19948 | 0.71958 | 0.277 | -1.21086548 | 1.609834597 | 0.78161 |
| Year (2019) | 0.03138 | 0.12050 | 0.260 | -0.20479335 | 0.267553208 | 0.79454 |
| Year (2020) | 0.01058 | 0.12716 | 0.083 | -0.23865274 | 0.259809222 | 0.93370 |
| Year (2021) | 0.43492 | 0.21497 | 2.023 | 0.01359644 | 0.856247360 | 0.04305 |
| Relative activity | 0.14017 | 0.05084 | 2.757 | 0.04051373 | 0.239817070 | 0.00584 |
| Life stage (Young) | -3.029 | 0.42517 | -7.123 | -3.86190412 | -2.195262254 | <0.001 |
|  | **Edf** | **Ref.df** | **Chi.sq** |  |  | **P** |
| Month (spline) | 6.958 | 8.001 | 224.2 |  |  | <0.001 |
| Site (random) | 42.773 | 52.000 | 817.7 |  |  | <0.001 |

**6 | Spearman Rank Correlation coefficients between all numerical predictor variables.**

**Table 6A:** Spearman Rank Correlation coefficients between all numerical predictor variables used within the ordinal Generalised Additive Model (GAM). Showing the strongest correlation between all continuous predictor variables was a moderate negative correlation between average annual rainfall (mm) and the mean maximum temperature at a site. This correlation did not meet the threshold (rho ≥ 0.7), so all predictor variables were retained in the analysis.

| **Numerical predictors** | **Month** | **Relative activity (PDE)** | **Average annual rainfall** | **Average maximum temperature** |
| --- | --- | --- | --- | --- |
| Month | 1 | 0.124476 | -0.01364 | 0.053541 |
| Relative activity (PDE) | 0.124476 | 1 | -0.07948 | 0.021254 |
| Average annual rainfall | -0.01364 | -0.07948 | 1 | -0.57835 |
| Average maximum temperature | 0.053541 | 0.021254 | -0.57835 | 1 |

**7 | Differences between adults and young with rumpwear: binomial regression and chi-square test of independence.**

**Table 7A:** Number of raw counts for each life-stage (adults and young) of brushtail possums. The observations were categorised into healthy (0), rumpwear (1), uncertain (2) or obscured (NA). Of the individuals for which we could see the rump, the percentage (%) is returned alongside the counts.

| **Rumpwear Score** | **Healthy** | **Uncertain** | **Rumpwear** | **Obscured** | **Total Assessments** |
| --- | --- | --- | --- | --- | --- |
| Adults | 5058 (84.2%) | 477 (7.9%) | 471 (7.8%) | 506 | 6512 |
| Young | 312 (98.1%) | 4 (1.3%) | 2 (0.6%) | 78 | 396 |
|  |  |  |  |  | 6908 |

**Table 7B:** The full dataset used below for the binomial logistic regression to establish if there was an age difference between adults and young displaying rumpwear (where 0 was healthy (no signs of rumpwear) and 1 was both rumpwear certain and uncertain signs). All adults and young were used in this analysis.

| **Life Stage** | **0 - healthy** | **1 – rumpwear or uncertain** |
| --- | --- | --- |
| Adult | 5058 | 948 |
| Juvenile | 312 | 6 |

**Table 7C:** The output for the binomial logistic regression between life-stages. We found statistical evidence that rumpwear (both certain and ambiguous signs) was associated with life-stage (LRT, G1 =67.47, P<0.001), where young brushtail possums were less likely to have rumpwear than adults (-2.28, 95% CI (-3.208, -1.557)).

|  | **DF** | **Deviance** | **LRT** | **Chi** |
| --- | --- | --- | --- | --- |
| None |  | 5297.7 |  |  |
| Life stage | 1 | 5365.1 | 67.468 | <0.001 |
|  |  |  |  |  |
| **Confidence Intervals** | **2.5%** | **97.5%** |  |  |
| Intercept | -1.744296 | -1.605548 |  |  |
| Life stage (juvenile) | -3.207650 | -1.557116 |  |  |
|  |  |  |  |  |
| **Coefficients** | **Estimate** | **Std. Error** | **z value** | **P** |
| Intercept | -1.67437 | 0.03539 | -47.310 | < 0.001 |
| Life stage (Juvenile) | -2.27687 | 0.41363 | -5.505 | < 0.001 |
|  |  |  |  |  |
| Null deviance: 5365.1 on 6323 degrees of freedom | | | | |
| Residual deviance: 5297.7 on 6322 degrees of freedom | | | | |
| (24 observations deleted due to missingness) | | | | |

**Table 7D:** The full output from the Chi-square test of independence (*X*^2^ [1, *N*= 790] = 48.7, *p* <.001). This was used to determine the relationship between actual and expected rumpwear scores for paired adults and young (n = 395). Young possums were less likely to have rumpwear.

| **Pearson's Chi-squared test:** Pairs of Life stage and rumpwear | | |
| --- | --- | --- |
| X-squared = 48.662 | df = 1 | p-value = 0.000000000003042 |
| **Expected Fit** |  |  |
|  | Rumpwear score |  |
| Life Stage | 0 (healthy) | 1 (rumpwear) |
| Adult | 307.5703 | 36.42965 |
| Juvenile | 283.4297 | 33.57035 |
| **Observed Fit** |  |  |
|  | Rumpwear score |  |
| Life Stage | 0 (healthy) | 1 (rumpwear) |
| Adult | 280 | 64 |
| Juvenile | 311 | 6 |

**8 | Output of the ordinal Generalised Additive Model on the predictors of rumpwear in adult brushtail possums.**

The coefficients and predicted probabilities from the model show the relative changes in the cumulative probabilities of the categories compared to the reference levels (-1 and 0). For example, a positive coefficient for a predictor indicates an increase in the cumulative probability of being in a higher category relative to the reference levels; while the effective degrees of freedom reflect the smoothness of the spline term and the reference degrees of freedom capture the complexity of the random effect variable.

| **Predictors** | **Estimate** | **Std. Error** | **z value** | **P** |
| --- | --- | --- | --- | --- |
| Intercept | -3.088157 | 0.251945 | -12.257 | < 0.001 |
| Rainfall | 0.054584 | 0.260182 | 0.210 | 0.83383 |
| Max Temperature | -0.189536 | 0.225826 | -0.839 | 0.40130 |
| Vegetation (N) | 0.195863 | 0.721059 | 0.272 | 0.78590 |
| Year (2019) | 0.016360 | 0.121099 | 0.135 | 0.89253 |
| Year (2020) | 0.007699 | 0.127739 | 0.060 | 0.95194 |
| Year (2021) | 0.436088 | 0.216210 | 2.017 | 0.04370 |
| Relative activity (PDE) | 0.137353 | 0.051089 | 2.688 | 0.00718 |
|  | **Edf** | **Ref.df** | **Chi.sq** | **P** |
| Month (spline) | 6.865 | 7.923 | 228.2 | <0.001 |
| Site (random) | 42.780 | 52.000 | 815.7 | <0.001 |

**Table 8A:** Coefficients and confidence intervals from the ordinal gam for each fixed effect. The coefficient estimates for each predictor variable represent the relative changes in the cumulative probabilities of the rumpwear levels (relative to the GAM reference levels of -1 and 0).

| **Predictors** | **coef** | **ci_lower (5)** | **ci_upper (95)** |
| --- | --- | --- | --- |
| (Intercept) | -3.08816 | -3.58196 | -2.59435 |
| Rainfall | 0.054584 | -0.45536 | 0.564531 |
| Temperature | -0.18954 | -0.63215 | 0.253076 |
| Vegetation | 0.195863 | -1.21739 | 1.609112 |
| year2019 | 0.01636 | -0.22099 | 0.253711 |
| year2020 | 0.007699 | -0.24266 | 0.258063 |
| year2021 | 0.436088 | 0.012324 | 0.859853 |
| Relative activity | 0.137353 | 0.037219 | 0.237487 |


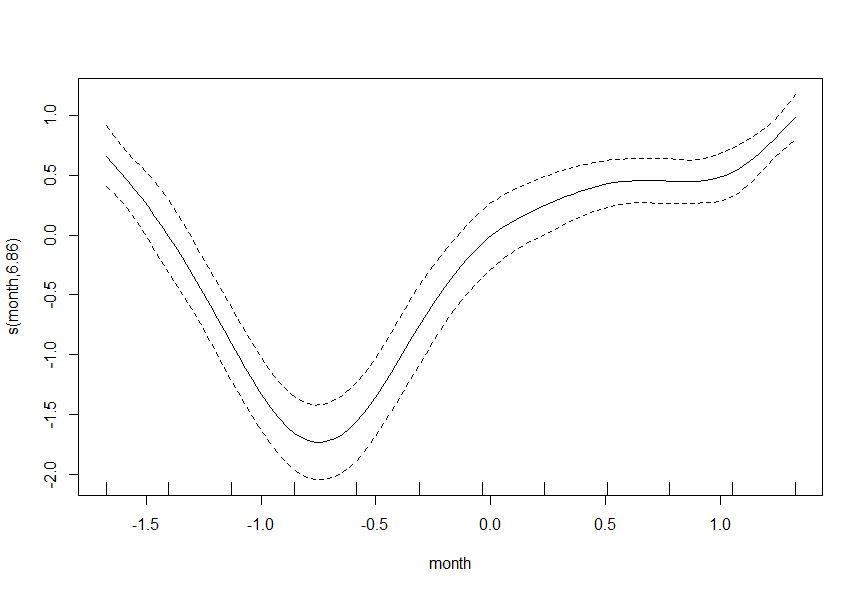


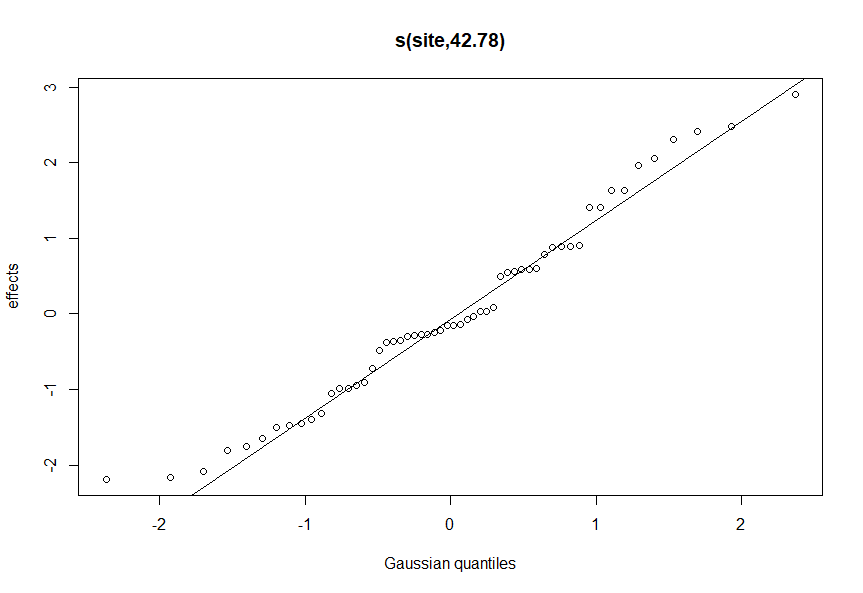
**Figure 8A:** Residual spline plot of month in the GAM model. The dashed lines represent the confidence intervals for the smooth terms, which are based on the effective degrees of freedom (EDF) rather than a standard error. The non-linear fit (solid black line) shows the relationship between the month and the log-odds of rumpwear (ordinal)

**Figure 8B:** Residual plot of the random effect of site in the ordinal GAM model. The straight black line represents the expected value of the random effect, which is centred around zero. The dotted points represent the individual site values. The y-axis ‘effects’ represents the estimated rumpwear values and the deviation from the overall average site level (i.e., whether a site had a positive or negative effect). The x-axis shows where the site sits along the standard deviations from the mean (-2 to3). The plot shows how the observed random effect values deviate from a normal distribution, allowing assessment of normality.


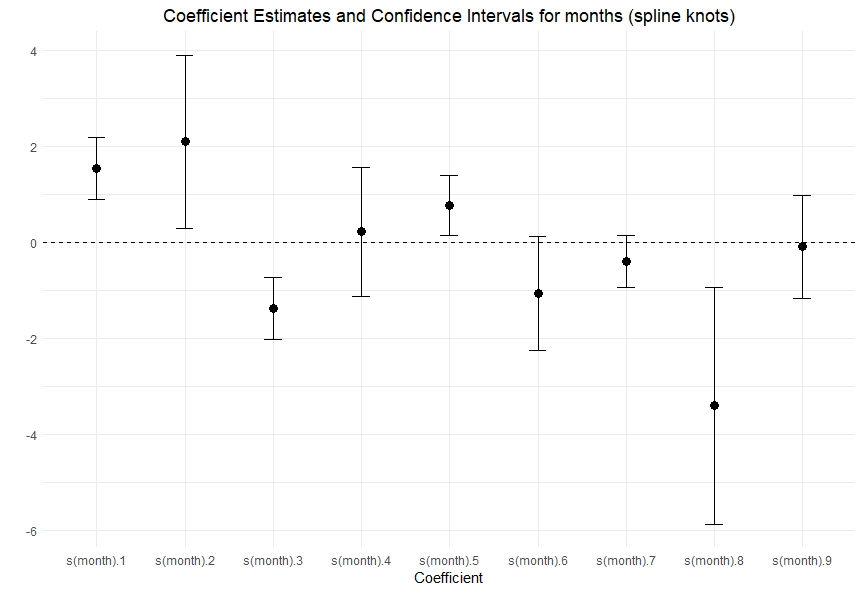


**Figure 8C:** Coefficient plot showing the estimate and 95% confidence intervals for each spline knot for the predictor ‘month’. If confidence intervals do not cross zero (dashed line), the knot has a significant influence on rumpwear in brushtail possums.


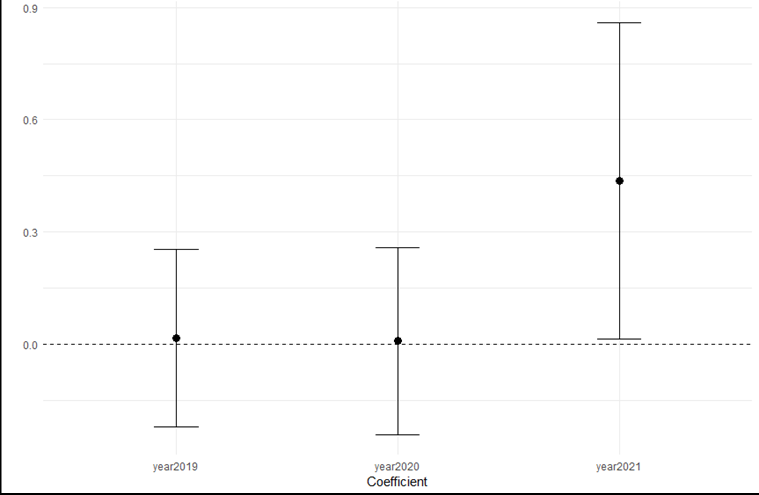


**Figure 8D:** The mixed-effect GAM coefficient plot showing the estimate and 95% confidence intervals for each year compared to 2018. The year 2021 confidence intervals do not cross zero (dashed line) and has a significant positive influence on the presence of rumpwear in brushtail possums compared to 2018.


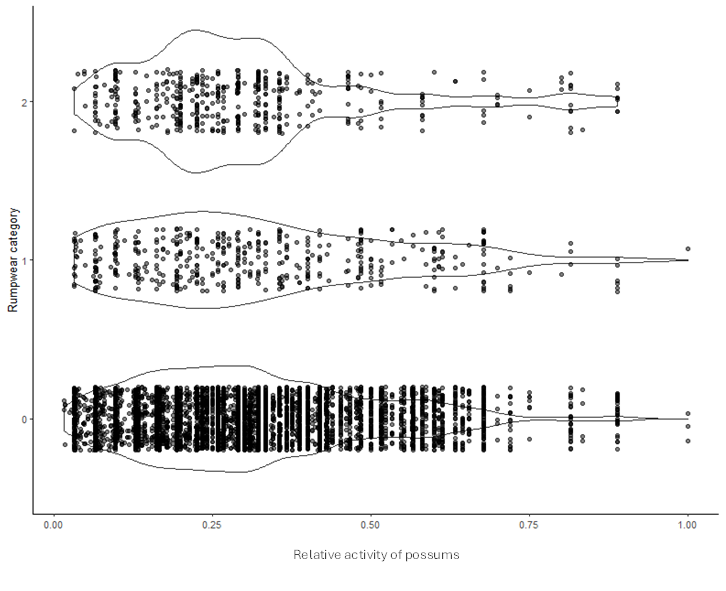


**Figure 8E**: Violin plot illustrating the distribution of relative activity of possums at a site per month for each rumpwear category (0 – healthy, 1 – uncertain, and 2 – rumpwear). Due to the high number of individual possums, the points overlap. The width of the violin indicates the frequency of datapoints within each rumpwear category. The 2 – rumpwear violin shows an increase in frequency at higher activity of possums in comparison to the other violins. The large number of data points enabled the GAM to detect statistical significance in the subtleties observed during visual inspection of the plot.


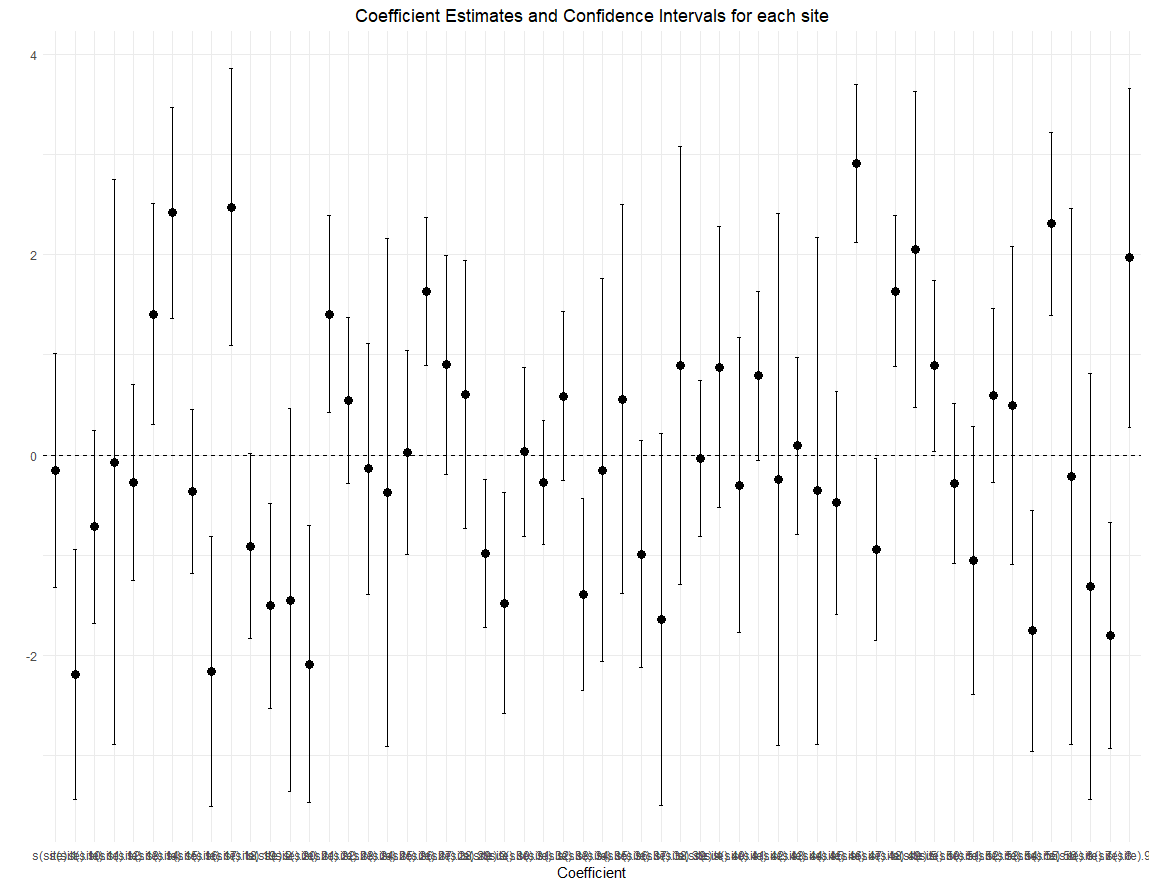


**Figure 8F:** Coefficient plot showing the estimate and 95% confidence intervals for each site compared to site one. If confidence intervals do not cross zero (dashed line), and therefore has a significant influence on the presence of rumpwear in brushtail possums.

**9 | Figures of the proportion of rumpwear** **in brushtail possums.**


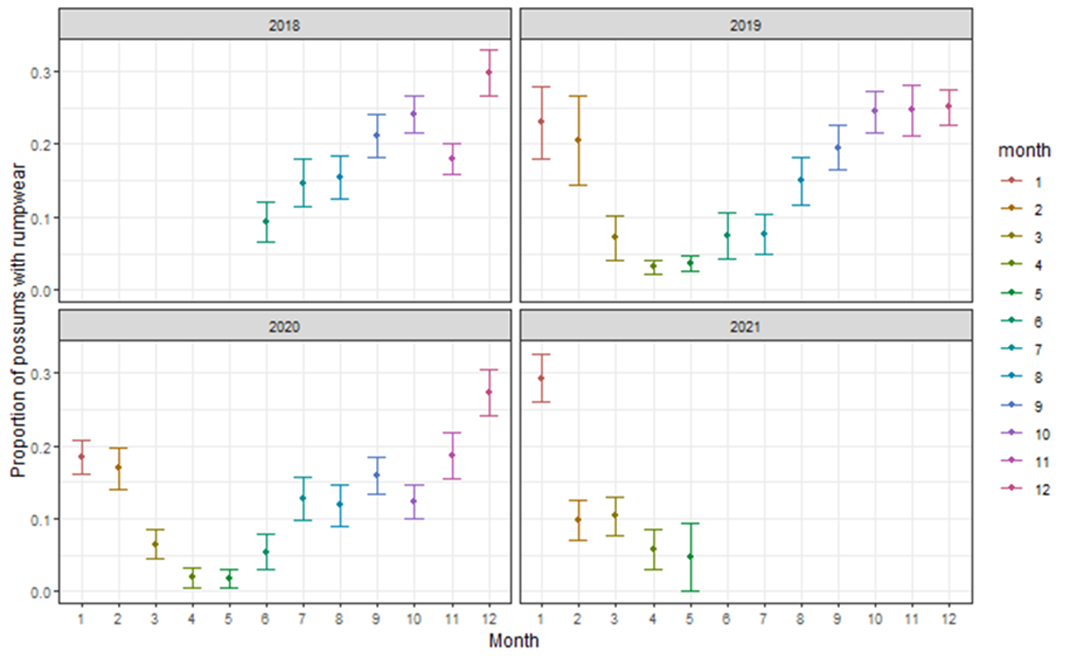


**Figure 9A:** Proportion of possums with rumpwear over month and year, showing standard error bars. The proportion is the number of possum events with rumpwear divided by the number of possum events in total (not including those for which rumpwear could not be observed (NA)).


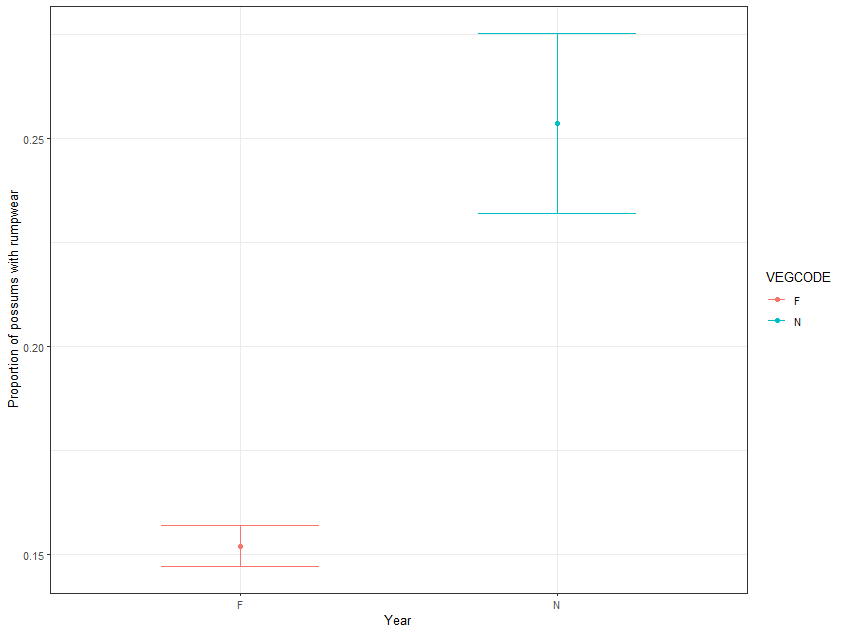


**Figure 9B:** Proportion of possums with rumpwear in each vegetation type, forest (F) or non-forest (N), showing standard error bars. Of the 57 sites, 49 sites were in the forest type, 8 were in the ‘non-forest’ type. There were 5828 healthy possums, 442 obscured (NAs), 819 possums with rumpwear recorded in the forest vegetation sites, and were 439 healthy possums, 33 obscured (NAs), and 103 possums with rumpwear recorded in the forest vegetation sites There is a difference between the vegetation types, with Non-forest having a higher proportion of rumpwear than forest, however they are not significantly different when compared in the GAM against the rest of the predictor variables.


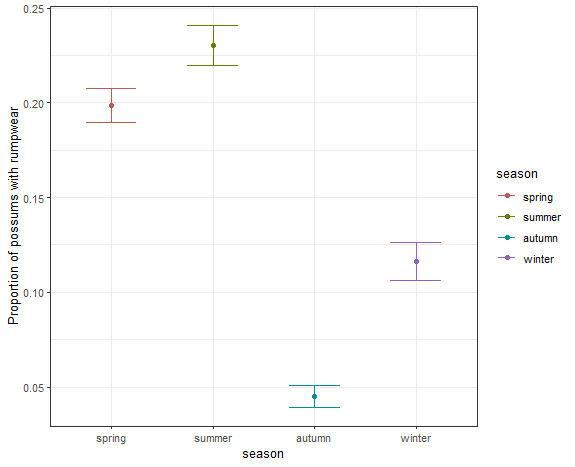


**Figure 9C:** Proportion of possums with rumpwear over each season, showing standard error bars. The temporal trend shows the Autumn months having lower prevalence of rumpwear (4.5%) in brushtail possums than other seasons (spring (19.9%), summer (23.0%), and winter (11.6%)).


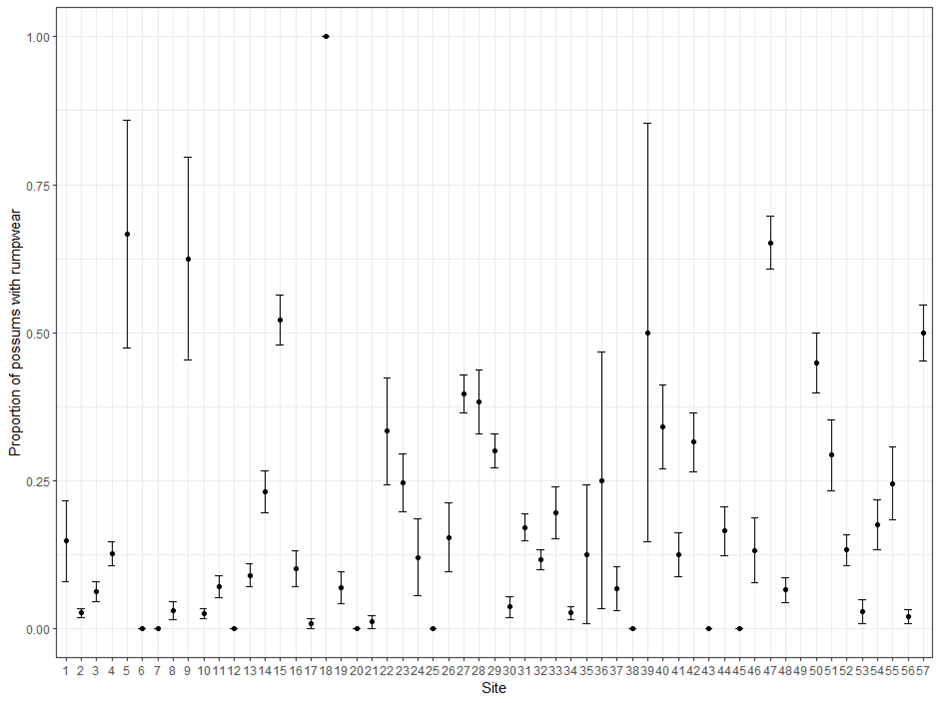


**Figure 9D:** The proportion of rumpwear at each site in the Adamsfield region. The proportion of possums with rumpwear included both rumpwear certain and uncertain categories (calculated: possums with rumpwear/total possums assessed), and standard error bars for each site. It shows there is variation in the proportion of possums with rumpwear at the sites.

**10 | Figures of component plots from GAM predictions.**


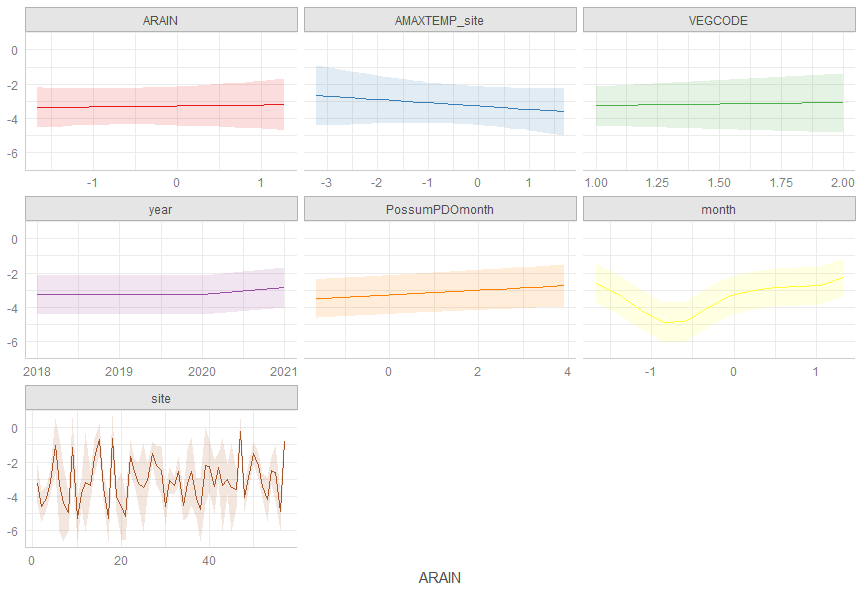
The component plot showing the relationship between the predictor variables (linear and non-linear) and rumpwear in the generalized additive model (GAM). The component plot was produced using the *ggeffects* package in R (Lüdecke, 2018) and computes the predicted log-odds of the response variable when holding all other predictors constant. The y-axis range of -6 to 0 represents the range of log-odds values for the predicted probabilities. Coloured Line: the predicted log-odds of the response variable. Shade: confidence intervals based on the standard errors for each predictor variable.

PDE

**11 |** **The convolution neural network (CNN) assessment of rumpwear in brushtail possums images across Tasmania.**


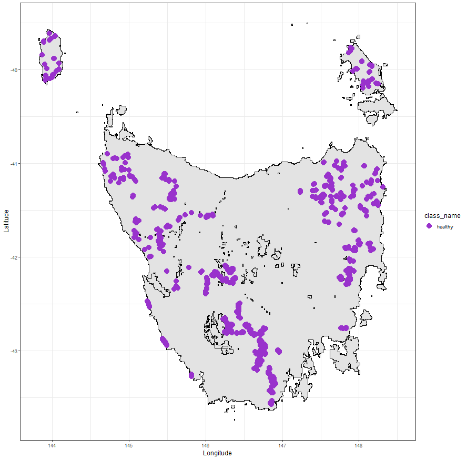

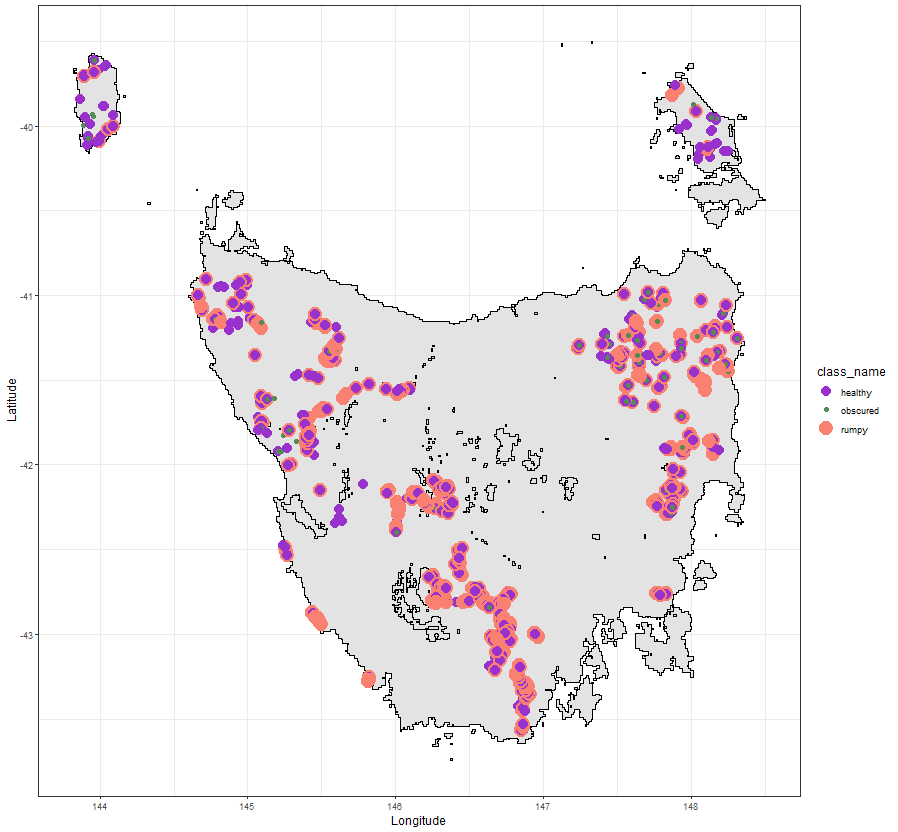


**(B)**


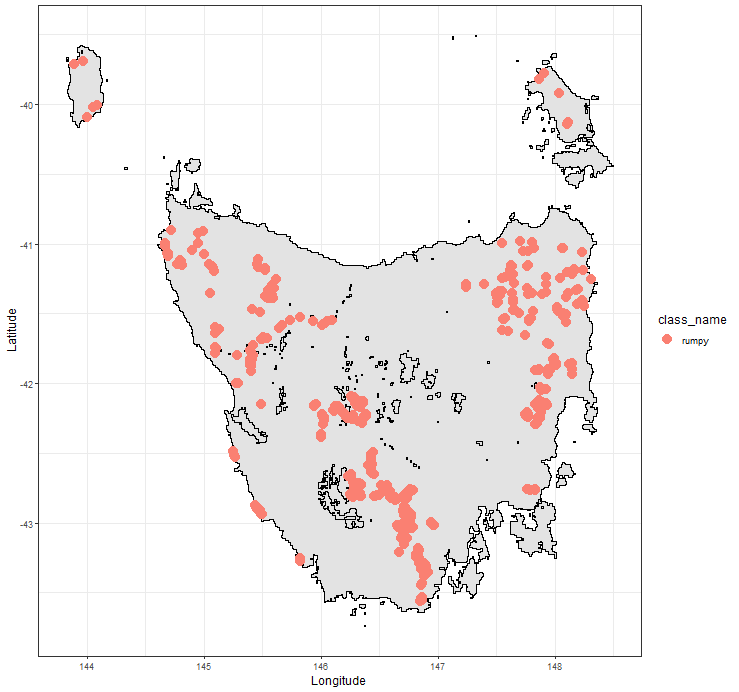


**(A)**

**(C)**

**Figure 11A**: The convolution neural network (CNN) predictions of rumpwear in brushtail possums from all 46,367 images (and no confidence threshold) across Tasmania, showing healthy (purple), obscured (green) and rumpwear (orange). **(A)** The distribution of possums, rumpwear and obscured images across the state; **(B)** images classified by CNN as healthy; and **(C)** images classified by CNN as rumpwear.
